# Supplementary material for: A basic framework to explain splice-site choice in eukaryotes
Source: Nat Commun. 2025 Sep 29;16:8284. doi: 10.1038/s41467-025-63622-9 (PMC12480690; doi:10.1038/s41467-025-63622-9)
Supplement: Supplementary file 1 — Supplementary Information File [file 41467_2025_63622_MOESM1_ESM.pdf]

Supplementary Material for

A basic framework to explain splice-site choice in eukaryotes

Craig I Dent[1,11, $], Stefan Prodic[1,12, $], Aiswarya Balakrishnan[1,13, $], Aaryan Chhabra[1], James D G Georges[1], Sourav Mukherjee[1], Jordyn Coutts[1], Michael Gitonobel[1], Rucha D Sarwade[1], Joseph Rosenbluh[2], Mauro D'Amato[3, 4, 5], Partha P Das[6, 7], Ya-Long Guo[8], Alexandre Fournier-Level[9], Richard Burke[1], Sridevi Sureshkumar[1], David Powell[10], and Sureshkumar Balasubramanian[1, *]

[1]School of Biological Sciences, Monash University, Clayton Campus, VIC 3800, Australia

[2]Department of Biochemistry and Molecular Biology and Cancer Program, Biomedicine Discovery Institute, Monash University, Clayton Campus, VIC 3800, Australia

[3]Department of Medicine and Surgery, LUM University, Casamassima, Italy

[4]Gastrointestinal Genetics Lab, CIC bioGUNE – BRTA, Derio, Spain

[5]Ikerbasque, Basque foundation for Science, Bilbao, Spain

[6]Department of Anatomy and Developmental Biology, Monash University, Clayton, VIC 3800, Australia

[7]Development and Stem Cells Program, Monash Biomedicine Discovery Institute, Clayton, VIC 3800, Australia.

[8]State Key Laboratory of Plant Diversity and Speciality Crops/State Laboratory of Systematic and Evolutionary Botany, Institute of Botany, Chinese Academy of Sciences, Beijing, 100093, China

[9]School of Biosciences, University of Melbourne, Parkville, Australia

[10]e-Research Centre, Monash University, Clayton Campus, VIC3800, Australia

[11]Present address: Max-Planck Institute for Plant Breeding Research, Cologne, Germany

[12]Present address: The Centre for Computational Biomedical Sciences, John Curtin School of Medical Research, Australian National University, Canberra, Australia

[13]Present address: University of Rochester Medical Center, University of Rochester, New York, USA

[$]These authors contributed equally – shared first authors.

[*]Author for correspondence

Sureshkumar Balasubramanian

School of Biological Sciences

Monash University

Clayton Campus

VIC 3800

AUSTRALIA

Email: mb.suresh@monash.edu

Species	Total number of sites	Total genes	Assessable sites	Assessable genes	>20% diff sites	% of sites	> 50% diff sites	% of sites
**Humans**	455744	14,733	391650	12658	324807	71.27	156370	34.31
**Arabidopsis**	219336	15099	112729	9804	54691	24.93	16860	7.69
**Drosophila(males)**	94095	9902	73130	8114	47888	50.89	10259	10.90
**Drosophila(females)**	78231	8197	65551	7215	33096	42.31	7030	8.99
**Total**	**769175**	**47931**	**643060**	**37791**	**427386**	**55.56**	**183489**	**23.86**

*** Drosophila numbers shown here are without filtering for replicates.**

Supplementary Table 1. A summary of the sites analyzed in Arabidopsis, Drosophila, and humans. Assessable sites refer to the number of sites for which SSE values could be calculated from at least 100 individuals. The number and percentage of splice-sites that show either 20% or 50% difference between individuals in their usage in Arabidopsis, Drosophila and Humans are shown.

Species	Site Chromosome	Site Position	Is it a clean Peak?	Is the top SNP the splice site?	Is the closest SNP the splice site?
Drosophila-Male	2L	3294119	Yes	No	Yes
Drosophila-Male	2L	3301757	Yes	Yes	Yes
Drosophila-Male	2L	3656838	Yes	No	Yes
Drosophila-Male	2L	5061319	Yes	No	No
Drosophila-Male	2L	6481970	Yes	No	Yes
Drosophila-Male	2L	7697158	Yes	Yes	Yes
Drosophila-Male	2L	9961618	Yes	Yes	Yes
Drosophila-Male	2L	13189690	Yes	Yes	Yes
Drosophila-Male	2L	14008436	Yes	Yes	Yes
Drosophila-Male	2L	16168472	Yes	Yes	Yes
Drosophila-Male	2L	18509779	Yes	No	No
Drosophila-Male	2L	20648188	Yes	No	No
Drosophila-Male	2L	20924168	Yes	No	Yes
Drosophila-Male	2R	5702294	Yes	Yes	Yes
Drosophila-Male	2R	7950439	Yes	No	Yes
Drosophila-Male	2R	8611102	Yes	No	Yes
Drosophila-Male	2R	9240686	Yes	Yes	Yes
Drosophila-Male	2R	10731658	Yes	No	Yes
Drosophila-Male	2R	12671475	Yes	Yes	Yes
Drosophila-Male	2R	15229627	Yes	No	No
Drosophila-Male	2R	15556015	Yes	Yes	Yes
Drosophila-Male	2R	15698848	Yes	No	Yes
Drosophila-Male	2R	15974719	No	No	No
Drosophila-Male	2R	15975698	Yes	No	No
Drosophila-Male	2R	15976072	Yes	No	No
Drosophila-Male	2R	15976088	Yes	No	No
Drosophila-Male	2R	17138259	Yes	No	No
Drosophila-Male	2R	17564718	Yes	Yes	Yes
Drosophila-Male	2R	18972998	No	No	No
Drosophila-Male	2R	19303342	No	No	No
Drosophila-Male	2R	19945010	Yes	No	No
Drosophila-Male	2R	22091250	Yes	Yes	Yes
Drosophila-Male	2R	22764807	Yes	Yes	Yes
Drosophila-Male	3L	1862064	Yes	No	Yes
Drosophila-Male	3L	4057485	No	No	No
Drosophila-Male	3L	15150711	Yes	Yes	Yes
Drosophila-Male	3L	15718090	No	No	No
Drosophila-Male	3L	15718935	Yes	Yes	Yes
Drosophila-Male	3L	16496052	Yes	Yes	Yes
Drosophila-Male	3L	18299651	Yes	No	No

Drosophila-Male	3L	19794700	Yes	No	Yes
Drosophila-Male	3L	20495055	Yes	No	Yes
Drosophila-Male	3L	21298778	Yes	Yes	Yes
Drosophila-Male	3R	9335496	No	No	No
Drosophila-Male	3R	12583230	Yes	No	Yes
Drosophila-Male	3R	14058131	Yes	No	No
Drosophila-Male	3R	14795745	Yes	No	No
Drosophila-Male	3R	18012095	Yes	Yes	Yes
Drosophila-Male	3R	21310999	No	No	No
Drosophila-Male	3R	21878171	Yes	No	Yes
Drosophila-Male	3R	25490627	Yes	Yes	Yes
Drosophila-Male	3R	26862772	No	No	No
Drosophila-Male	3R	28836686	Yes	Yes	Yes
Drosophila-Male	3R	28885710	Yes	No	No
Drosophila-Male	3R	29244448	No	No	No
Drosophila-Male	3R	30212454	Yes	No	Yes
Drosophila-Male	3R	30705990	Yes	Yes	Yes
Drosophila-Male	X	2071861	Yes	Yes	Yes
Drosophila-Male	X	9609750	Yes	Yes	Yes
Drosophila-Male	X	14825956	Yes	Yes	Yes
Drosophila-Male	X	18896382	No	No	No
Drosophila-Male	X	21046757	Yes	No	Yes
Drosophila-Male	X	21047118	Yes	Yes	Yes
Drosophila-Female	2L	3301757	Yes	Yes	Yes
Drosophila-Female	2L	5061319	No	No	No
Drosophila-Female	2L	6481970	Yes	No	No
Drosophila-Female	2L	7697158	No	No	No
Drosophila-Female	2L	9961618	Yes	Yes	Yes
Drosophila-Female	2L	13189690	Yes	Yes	Yes
Drosophila-Female	2L	18509779	Yes	No	No
Drosophila-Female	2L	20648188	Yes	No	No
Drosophila-Female	2L	22538626	Yes	Yes	Yes
Drosophila-Female	2R	5702294	Yes	Yes	Yes
Drosophila-Female	2R	15556015	Yes	Yes	Yes
Drosophila-Female	2R	15974719	No	No	No
Drosophila-Female	2R	15975698	Yes	No	No
Drosophila-Female	2R	15976072	Yes	No	No
Drosophila-Female	2R	15976088	Yes	No	No
Drosophila-Female	2R	17138259	Yes	No	No
Drosophila-Female	2R	19303342	No	No	No
Drosophila-Female	2R	22092063	No	No	No
Drosophila-Female	3L	1658125	Yes	Yes	Yes
Drosophila-Female	3L	1862064	Yes	No	Yes

Drosophila-Female	3L	16496052	Yes	Yes	Yes
Drosophila-Female	3L	18841151	Yes	Yes	Yes
Drosophila-Female	3R	9335496	Yes	Yes	Yes
Drosophila-Female	3R	14795745	Yes	No	No
Drosophila-Female	3R	15955935	Yes	Yes	Yes
Drosophila-Female	3R	21310999	Yes	No	No
Drosophila-Female	3R	25490627	Yes	Yes	Yes
Drosophila-Female	3R	26862772	No	No	No
Drosophila-Female	3R	28836686	Yes	No	No
Drosophila-Female	3R	28885710	Yes	No	No
Drosophila-Female	X	9609750	Yes	Yes	Yes
Drosophila-Female	X	14825956	No	No	No
Drosophila-Female	X	21046757	Yes	No	Yes
Drosophila-Female	X	21047118	Yes	Yes	Yes
Humans	chr1	66776404	Yes	No	Yes
Humans	chr1	172462246	Yes	Yes	Yes
Humans	chr1	179889309	Yes	Yes	Yes
Humans	chr10	73126634	No	No	No
Humans	chr10	97679916	Yes	Yes	Yes
Humans	chr11	47237713	Yes	No	Yes
Humans	chr11	61398269	Yes	No	Yes
Humans	chr11	65859230	No	No	No
Humans	chr11	113368360	No	No	Yes
Humans	chr12	5929994	Yes	No	No
Humans	chr12	31117654	No	No	No
Humans	chr13	52143914	Yes	Yes	Yes
Humans	chr14	31389003	Yes	No	Yes
Humans	chr14	73245245	No	No	No
Humans	chr14	77507610	No	No	No
Humans	chr14	104115323	Yes	Yes	Yes
Humans	chr15	40564790	Yes	Yes	Yes
Humans	chr16	15882948	Yes	No	Yes
Humans	chr17	317099	No	No	No
Humans	chr17	69179009	Yes	No	Yes
Humans	chr17	69223627	No	Yes	Yes
Humans	chr17	75590244	Yes	Yes	Yes
Humans	chr18	2540666	No	No	No
Humans	chr18	11905954	Yes	Yes	Yes
Humans	chr19	15678330	Yes	No	Yes
Humans	chr19	47349962	No	No	No
Humans	chr19	57492212	Yes	Yes	Yes
Humans	chr2	169584491	Yes	Yes	Yes
Humans	chr2	204570848	Yes	No	No

Humans	chr20	35626801	Yes	Yes	Yes
Humans	chr3	75784914	No	No	No
Humans	chr3	131387187	Yes	Yes	Yes
Humans	chr3	183636220	Yes	Yes	Yes
Humans	chr3	196249998	No	No	No
Humans	chr4	99879876	Yes	No	No
Humans	chr4	140524662	No	No	No
Humans	chr4	168396128	Yes	Yes	Yes
Humans	chr5	83353124	Yes	Yes	Yes
Humans	chr5	177714053	Yes	No	Yes
Humans	chr6	2721715	No	No	No
Humans	chr6	26368051	No	No	No
Humans	chr6	31810495	No	No	No
Humans	chr7	93261814	No	No	No
Humans	chr7	158871607	Yes	Yes	Yes
Humans	chr8	123142457	Yes	Yes	Yes
Humans	chr9	91852320	Yes	Yes	Yes
Humans	chrX	1298749	No	No	No
Humans	chrX	153674273	No	No	No
Arabidopsis	1	519035	Yes	Yes	Yes
Arabidopsis	1	1399466	Yes	No	Yes
Arabidopsis	1	2026756	No	No	No
Arabidopsis	1	3198442	No	No	No
Arabidopsis	1	4200107	Yes	Yes	Yes
Arabidopsis	1	4593559	No	No	No
Arabidopsis	1	8200719	Yes	No	No
Arabidopsis	1	12145683	Yes	No	Yes
Arabidopsis	1	17973080	Yes	Yes	Yes
Arabidopsis	1	19523303	Yes	Yes	Yes
Arabidopsis	1	20077746	No	No	No
Arabidopsis	1	21763877	Yes	No	Yes
Arabidopsis	1	21982990	Yes	No	No
Arabidopsis	1	22983032	Yes	Yes	Yes
Arabidopsis	1	25467618	Yes	No	No
Arabidopsis	2	634082	Yes	No	No
Arabidopsis	2	7685018	No	No	No
Arabidopsis	2	10452420	Yes	No	No
Arabidopsis	2	11816928	Yes	Yes	Yes
Arabidopsis	2	13659293	Yes	Yes	Yes
Arabidopsis	2	13663606	No	No	No
Arabidopsis	2	17345987	No	No	No
Arabidopsis	2	17403809	Yes	No	Yes
Arabidopsis	2	19065947	Yes	No	No

Arabidopsis	3	300406	Yes	Yes	Yes
Arabidopsis	3	6429309	Yes	No	Yes
Arabidopsis	3	7424535	Yes	No	Yes
Arabidopsis	3	8421655	Yes	No	No
Arabidopsis	3	8422269	Yes	Yes	Yes
Arabidopsis	3	11291361	Yes	No	Yes
Arabidopsis	3	11979038	Yes	Yes	Yes
Arabidopsis	3	21829914	Yes	Yes	Yes
Arabidopsis	3	22728605	Yes	Yes	Yes
Arabidopsis	4	2418449	No	No	No
Arabidopsis	4	2718912	Yes	Yes	Yes
Arabidopsis	4	5385280	Yes	Yes	Yes
Arabidopsis	4	7843450	Yes	No	Yes
Arabidopsis	4	7892649	No	No	No
Arabidopsis	4	8287347	Yes	Yes	Yes
Arabidopsis	4	12610362	Yes	Yes	Yes
Arabidopsis	4	12865306	Yes	Yes	Yes
Arabidopsis	4	14167974	Yes	No	No
Arabidopsis	4	15104520	Yes	No	Yes
Arabidopsis	4	16353607	Yes	Yes	Yes
Arabidopsis	4	17229824	No	No	No
Arabidopsis	4	17645733	No	No	No
Arabidopsis	5	3719684	No	No	No
Arabidopsis	5	4727093	Yes	No	No
Arabidopsis	5	5404673	Yes	No	No
Arabidopsis	5	6022916	Yes	Yes	Yes
Arabidopsis	5	7517704	No	No	No
Arabidopsis	5	7927662	Yes	No	Yes
Arabidopsis	5	14237725	Yes	Yes	Yes
Arabidopsis	5	17843414	Yes	No	No
Arabidopsis	5	18757786	Yes	No	Yes
Arabidopsis	5	19125980	No	No	No
Arabidopsis	5	24763662	Yes	No	Yes
Arabidopsis	5	25985653	Yes	No	Yes

Supplementary Table 2. SpliSER-GWAS results for all "GWASable" splice-site mutations in Arabidopsis, Drosophila and Humans.

	Author	Year	Journal	Phenotype	Genetic mapping	Method	Species	Tissues	Minimum Samples	Range of testing	Can it detect Trans?	Total sQTLs
1	Garrido-Martin et al	2021	Nature Communications	Isoform abundances	Yes	sQTLseekeR 2	Human	Multiple	70 or more	plu/minus 5Kb	NO	210,485
2	Qi et al	2022	Nature Genetics	Isoform abundances	Yes	THISTLE	Human	Multiple	No filtering	plus/minus 2Mb	NO	795,592
						sQTLseekeR						390,497
3	GTEx consortium	2020	Science	Intron excision ratio	Yes	FastQTL	Human	Multiple		plus/minus 1Mb	NO	
4	Li et al	2018	Nature Genetics	Intron excision ratio	Yes			Four tissues		plus/minus 50Kb	NO	442
5	Khokhar	2019	Frontiers in plant science	splicing ratios	Yes	UlfasQTL (sQTLseekeR)	Arabidopsis	single mix	No filtering	Genome	YES	6,406
**6**	**Dent et al**	**2024**		**Splice-site strength (SSE)**	**Yes**	**GWAS**	**Human**	**Heart**	**100**	**Genome**	**YES**	**9872**
						**GWAS**	**Arabidopsis**	**Mix**	**100**	**Genome**	**YES**	**5277**
						**GWAS**	**Drosophila**	**males**	**100**	**Genome**	**YES**	**3897**
						**GWAS**	**Drosophila**	**females**	**100**	**Genome**	**YES**	**3453**
7	Barbosa-Morais et al	2012	Science	PSI values for exons	No		Multiple	Multiple			NO	
8	Merkin et al	2012	Science	PSI values for exons	No		Multiple	Multiple			NO	

Supplementary Table 3. A comparative summary of previous sQTL analysis vs SpliSER-GWAS. SpliSER-GWAS study section is in bold.

Number	Species	Donor/Acceptor	Total Splice sites	Hexamer Rank 1 Sites	Percentage of sites explained by hexamer ranks
1	Human	Donors	182512	139923	76.67
2	Human	Acceptors	169860	103513	60.94
3	Drosophila	Donors	41983	35430	84.39
4	Drosophila	Acceptors	39399	29100	73.86
5	Arabidopsis	Donors	92246	55854	60.55
6	Arabidopsis	Acceptors	85663	60120	70.18
7	Rice	Donors	64898	37759	58.18
8	Rice	Acceptors	62030	43793	70.60
9	C elegans	Donors	1709	726	42.48
10	C elegans	Acceptors	1739	1444	83.04
11	Chicken	Donors	83736	64999	77.62
12	Chichen	Acceptors	80648	51581	63.96
13	Chimp	Donors	117165	92195	78.69
14	Chimp	Acceptors	114214	70227	61.49
15	Cobra	Donors	47319	34895	73.74
16	Cobra	Acceptors	47447	29579	62.34
17	Corn	Donors	46225	32147	69.54
18	Corn	Acceptors	47461	26163	55.13
19	Opium	Donors	18064	5102	28.24
20	Opium	Acceptors	18306	11404	62.30
21	Pig	Donors	14520	6723	46.30
22	Pig	Acceptors	17312	6802	39.29
23	Potato	Donors	47503	26248	55.26
24	Potato	Acceptors	47151	31856	67.56
25	Sorghum	Donors	76514	46476	60.74
26	Sorghum	Acceptors	72794	50963	70.01
27	Tomato	Donors	38109	18849	49.46
28	Tomato	Acceptors	36629	25480	69.56
29	Xenopus	Donors	122954	96211	78.25
30	Xenopus	Acceptors	119674	78537	65.63
31	ZebraFish	Donors	117839	76586	64.99
32	ZebraFish	Acceptors	113729	67856	59.66
33	Canola	Donors	173671	112532	64.80
34	Canola	Acceptors	168594	117280	69.56
35	Tegu	Donors	101340	80184	79.12
36	Tegu	Acceptors	97615	61826	63.34
37	Hydra	Donors	73004	30851	42.26
38	Hydra	Acceptors	71118	51566	58.12
39	Chara	Donors	4642	2675	57.63
40	Chara	Acceptors	4716	2900	61.49

Supplementary Table 4. Hexamer ranking explains most of the splice-site choice across the genome in diverse organisms. Here, percentage explained refers to the

number of splice-sites with highest average strength hexamer in a window of +/-50bp without applying a penalty for duplication.

Oligo	Gene	Sequence	Purpose
OSKB_4940	AT1G21920	CAA TCC CGG TTT AGC TTT CA	To verify GWAS cis associations
OSKB_4941	AT1G21920	TTC TCC GGT TTT GAA TCG TC	To verify GWAS cis associations
OSKB_4942	AT5G42900	AAT CCC GGT AAC GGA TGA G	To verify GWAS cis associations
OSKB_4943	AT5G42900	GAT CGG TAC TCG ACG ATT CA	To verify GWAS cis associations
OSKB_4944	AT1G28520	GGA GTA GGT GAA GAG TTT AAC TAT CTG	To verify GWAS cis associations
OSKB_4945	AT1G28520	TAA CTC CAG GAC AGT CTT CAG C	To verify GWAS cis associations
OSKB_4946	AT2G44680	TTC TGT TGC GGA CAG TCT TG	To verify GWAS cis associations
OSKB_4947	AT2G44680	AAA CCC GTG ACC ATC AGA AG	To verify GWAS cis associations
OSKB_5159	AT1G31910	GCA ACC GAG AAG GAC AAA GA	To verify GWAS trans associations
OSKB_5160	AT1G31910	GGC TTG GAA TTA GCA CCA TT	To verify GWAS trans associations
OSKB_5630	mCHERRY	TCA AGC CTC AGA CAG TGG TTC	To verify pJG inserts in mCHERRY via sequencing
OSKB_5631	mCHERRY	CAT AGC GTA AAA GGA GCA ACA	To verify pJG inserts in mCHERRY via sequencing
OSKB_5658	mCHERRY	CCT GCA GGA CGG CGA GTT CAT	To verify hexamer ranking based splice-site utilisation and sequencing
OSKB_5659	mCHERRY	GAA GTT GGT GCC GCG CAG CTT	To verify hexamer ranking based splice-site utilisation and sequencing
OSKB_5715	mCHERRY	CCG ACA TCC CCG ACT ACT TGA	To verify hexamer ranking based splice-site utilisation and sequencing
OSKB_5716	mCHERRY	CTG CTT GAT CTC GCC CTT CAG	To verify hexamer ranking based splice-site utilisation and sequencing

Supplementary Table 5. Primers used in this study.

Tel: +61 3 99051373

Supplementary Figures

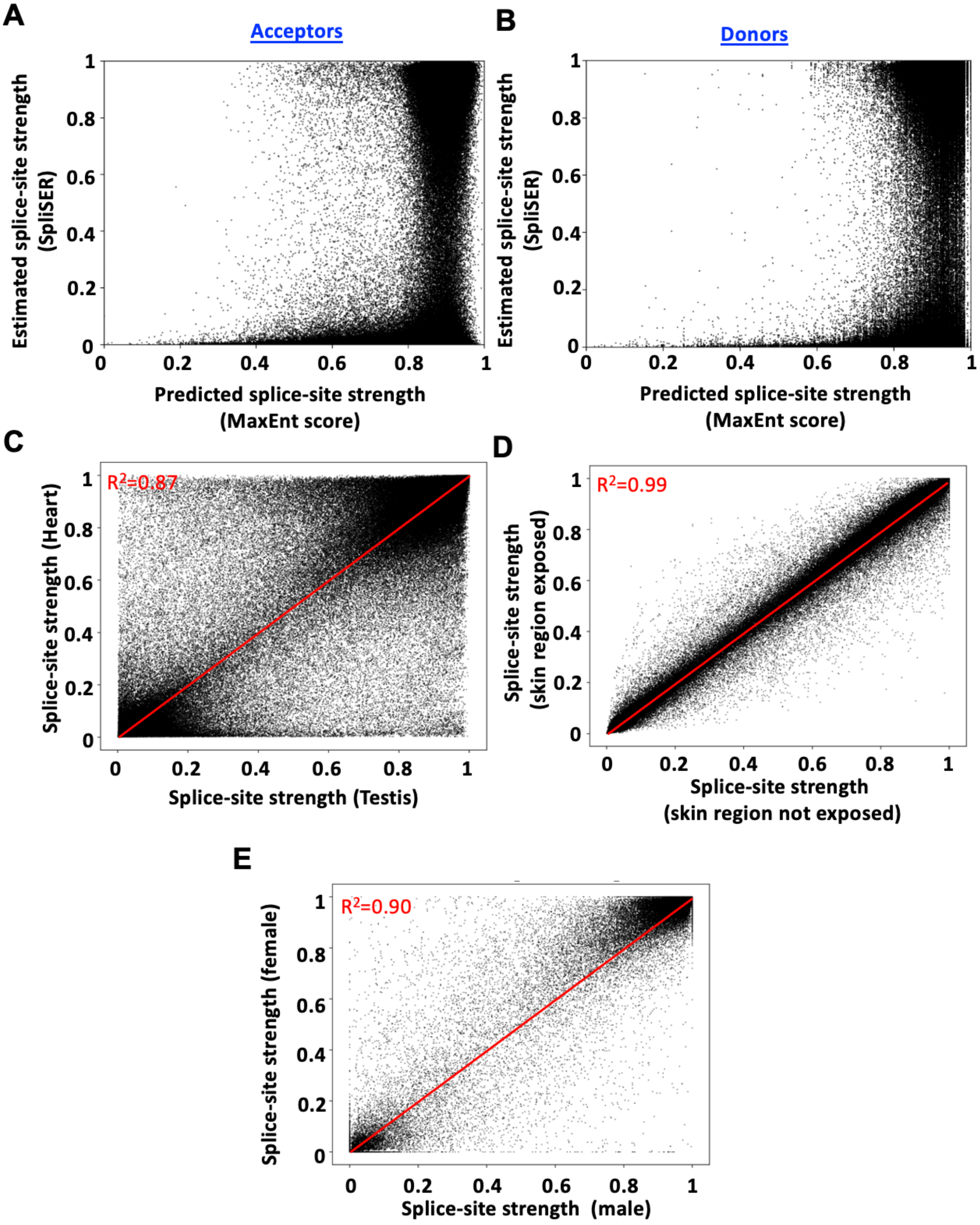

Supplementary Figure 1. Empirical quantifications by SpliSER differ noticeably from MaxEnt predictions of splice-site strength and allow detecting context-dependent variability in splice-site usage. A-B) Correlation between empirical estimations of splice-site strength by SpliSER and MaxEnt scores for acceptors (A) and donors (B). MaxEnt scores are scaled in the range of 0 to 1 for this analysis. C) Correlation of splice-site strengths between common sites detected in human heart and testis. D) Correlation of splice-site strengths between sites in two different skin tissues. E) Correlation of splice-site strengths between sites in male and female drosophila samples.

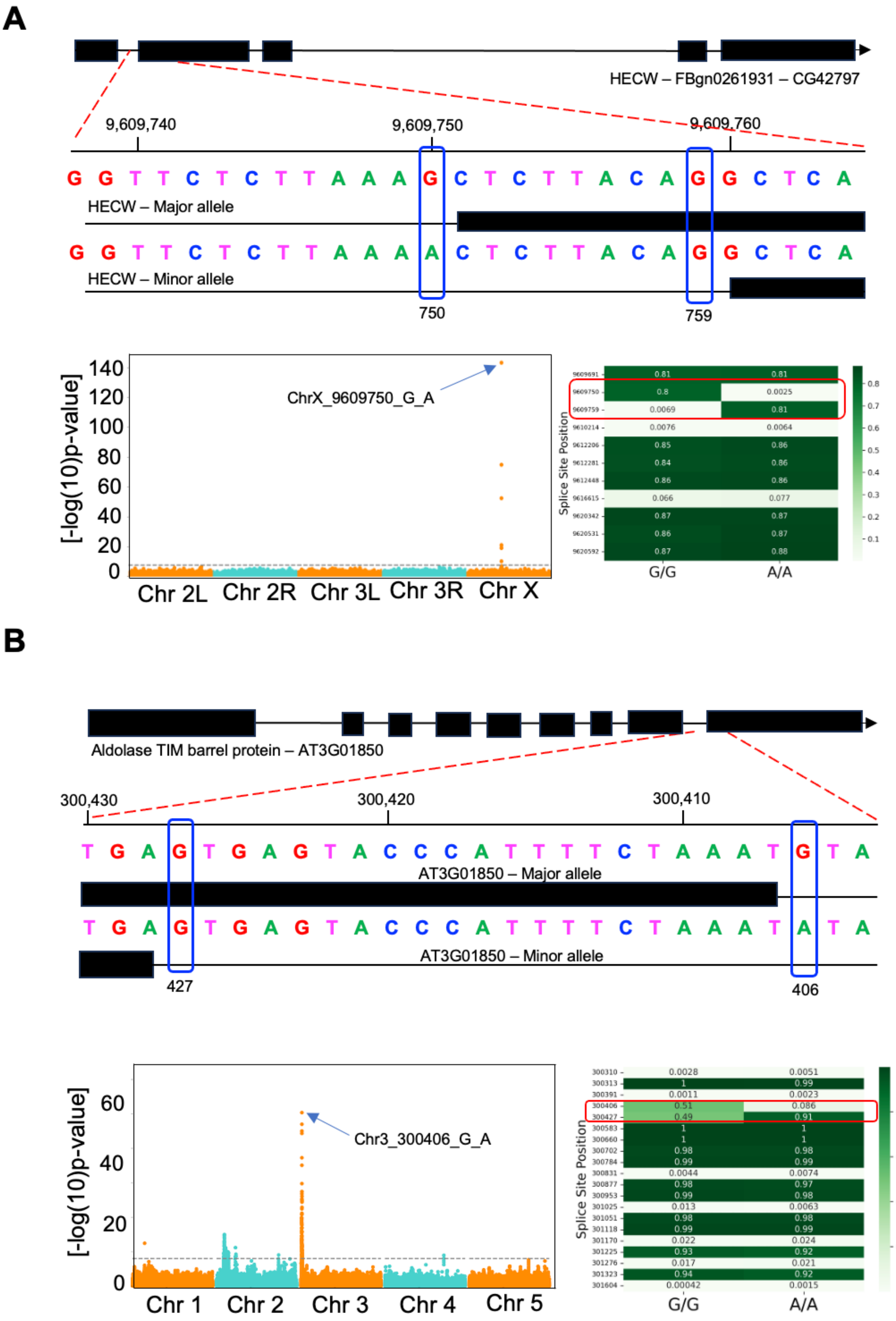
A
HECW – FBgn0261931 – CG42797
9,609,740
9,609,750
9,609,760
HECW – Major allele
HECW – Minor allele
750
759
[-log(10)p-value]
ChrX_9609750_G_A
Chr 2L
Chr 2R
Chr 3L
Chr 3R
Chr X
Splice Site Position
G/G
A/A
B
Aldolase TIM barrel protein – AT3G01850
300,430
300,420
300,410
AT3G01850 – Major allele
AT3G01850 – Minor allele
427
406
Chr3_300406_G_A
Chr 1
Chr 2
Chr 3
Chr 4
Chr 5

Supplementary Figure 2. Variation in splice-site usage can be mapped accurately with SpliSER-GWAS. A) Manhattan plot of the for the splice-site mutation at in the Drosophila *HECW* gene. SpliSER-GWAS analysis identifies causal SNP for variation in the usage of splice-sites at the *HECW* locus in Drosophila. A schematic of the sequences surrounding two competing splice-sites at the *HECW* gene. A mutation in the *HECW* gene (Fbgn0261931-CG42797) of Drosophila at position chrX:9,609,750 (750) abolishes a splice acceptor site that leads to an increased usage of a site at chrX:9,609,759 (759). Average splice-site usage of the sites 750 and 759 and the neighboring splice-sites. The effect of mutation at 750 is specific to 750 and 759 and does not affect neighboring sites. B). A schematic of sequences surrounding two competing sites at AT3G01850. The splice-site mutation at 406 allows the usage of 427 as a splice-site, and variation in the usage of 406 as well as 427 maps to the 406 polymorphism. SpliSER-GWAS identifies chr3:300406 as the causal SNP for variation in the usage of splice-sites at the AT3G01850 locus in Arabidopsis. Average splice site usage of the sites 427 and 406. The effect of the mutation is specific to 427 and 406 and does not affect neighboring sites.

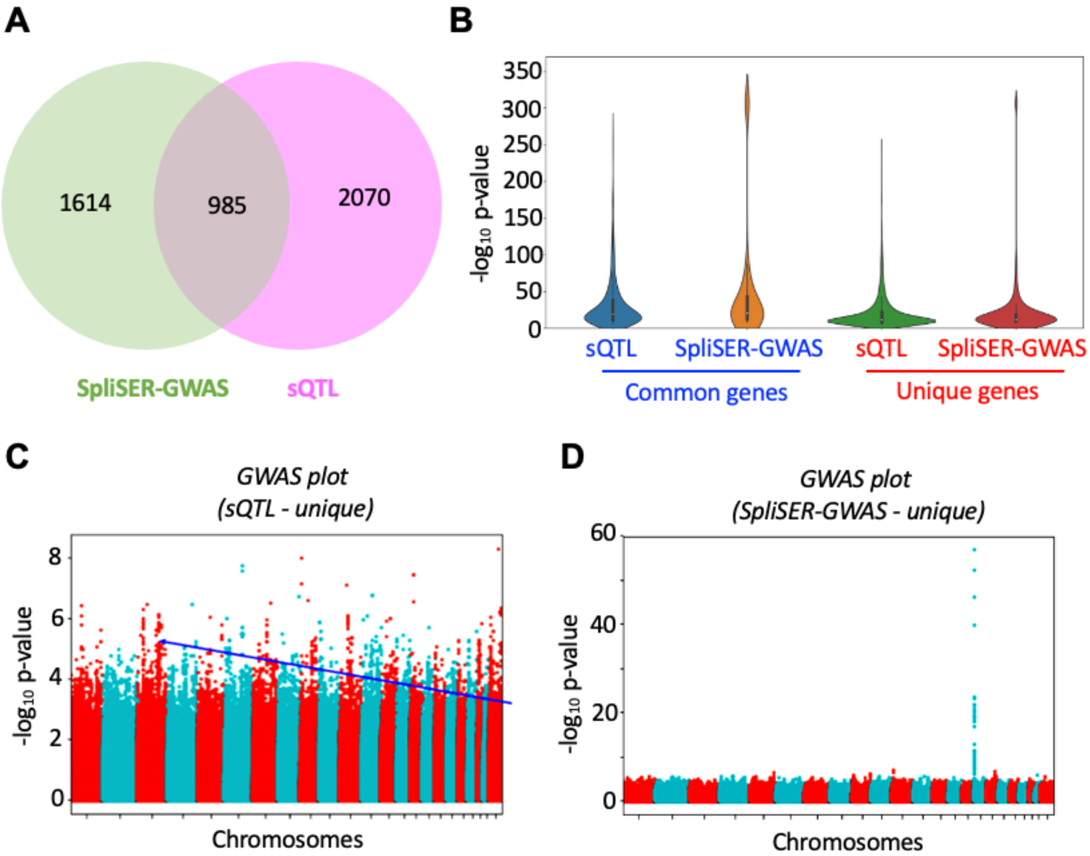

Supplementary Figure 3. SpliSER-GWAS is complementary and captures significant variation missed by sQTL analysis. A) Common and unique genes captured via SpliSER-GWAS and sQTL analysis. B) Distribution of the negative log p-values of significant SNPs for common genes and unique genes in sQTL analysis and SpliSER-GWAS. The lowest p-values in SpliSER-GWAS are given the same value, which is giving a bulged peak appearance. C) GWAS plot of a site in a gene that is uniquely captured by sQTL analysis. The blue arrow shows the SNP that was identified to be the significant SNP in the sQTL analysis. D) GWAS plot for a site from a unique gene captured by SpliSER-GWAS.

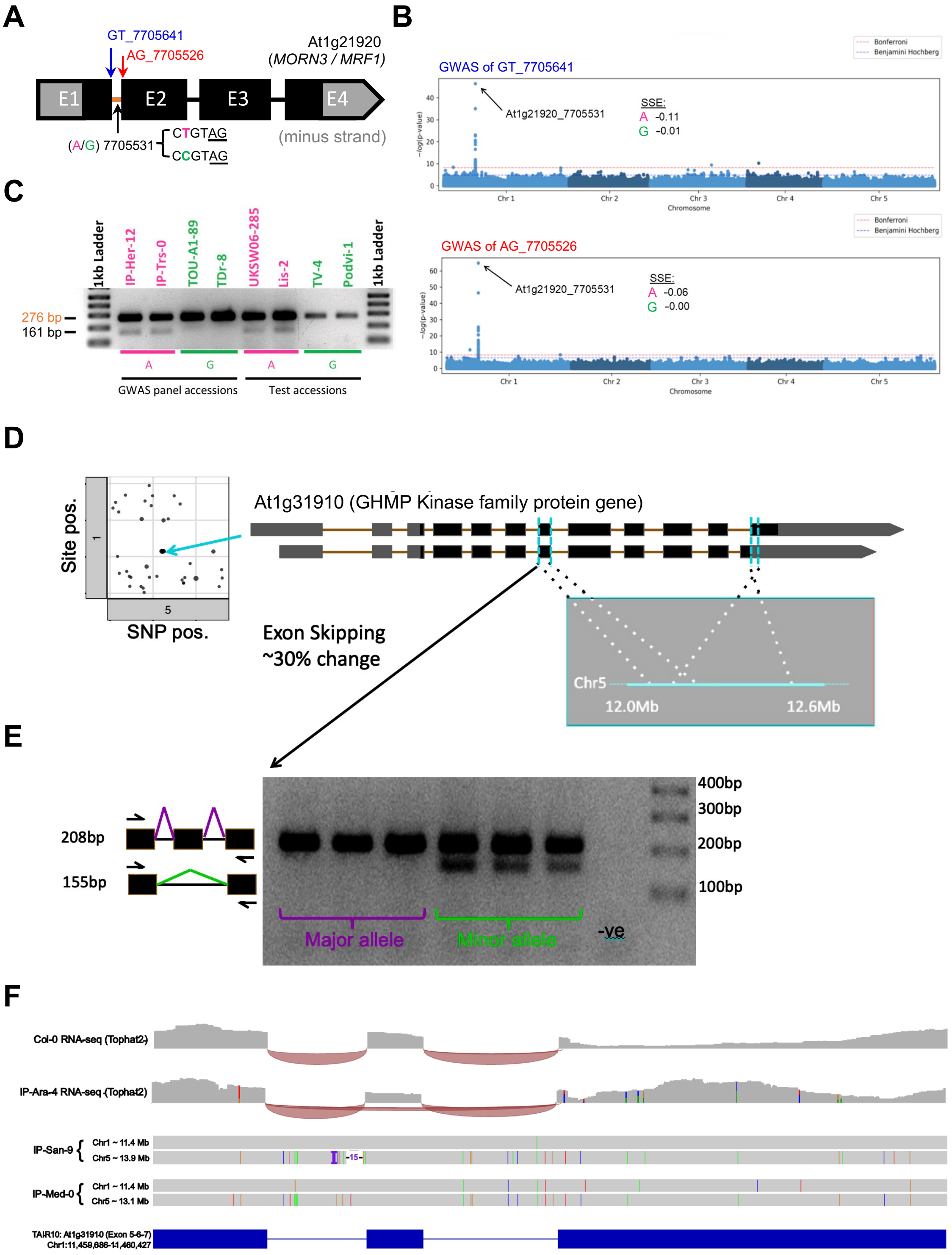
A
GT_7705641
AG_7705526
At1g21920
(MORN3 / MRF1)
E1
E2
E3
E4
(A/G) 7705531
CTGTAG
CCGTAG
(minus strand)
B
GWAS of GT_7705641
At1g21920_7705531
SSE:
A -0.11
G -0.01
Bonferroni
Benjamini Hochberg
GWAS of AG_7705526
At1g21920_7705531
SSE:
A -0.06
G -0.00
Chromosome
C
1kb Ladder
IP-Her-12
IP-Trs-0
TOU-A1-89
TDr-8
UKSW06-285
Lis-2
TV-4
Podvi-1
276 bp
161 bp
GWAS panel accessions
Test accessions
D
Site pos.
SNP pos.
At1g31910 (GHMP Kinase family protein gene)
Exon Skipping
~30% change
Chr5
12.0Mb
12.6Mb
E
208bp
155bp
400bp
300bp
200bp
100bp
Major allele
Minor allele
-ve
F
Col-0 RNA-seq (Tophat2)
IP-Ara-4 RNA-seq (Tophat2)
IP-San-9
Chr1 ~ 11.4 Mb
Chr5 ~ 13.9 Mb
IP-Med-0
Chr1 ~ 11.4 Mb
Chr5 ~ 13.1 Mb
TAIR10: At1g31910 (Exon 5-6-7)
Chr1:11,459,686-11,460,427

Supplementary Figure 4. SpliSER-GWAS associations are experimentally verifiable. A-C) Mapping of a minor-effect intron splicing at AT1G21920, which encodes a histone methyl transferase and natural variation of an SNP at position 7705531 is associated with the use of both splice-donor and acceptor sites. **C**) The presence of “G” at this position abolishes splicing (larger bands) and presence of “A” leads to partial splicing of the intron resulting in two bands. Test accessions that are not part of the GWAS panel display the same splicing patterns confirming GWAS associations. D) Experimental verification of a *trans* association in *Arabidopsis thaliana.* An apparent exon-skipping outcome that occurs due to differential usage of few different sites in a gene in Chromosome 1 that encodes a GHMP kinase family protein maps to a region in Chromosome 5. Several *trans* associations map to the same region. **E)** RT-PCR analysis with specific genotypes confirmed the association. **F)** long-read genome assemblies revealed that there is an inter chromosomal gene duplication that could best explain the observed variation, which may be caused potentially due to mapping issues.

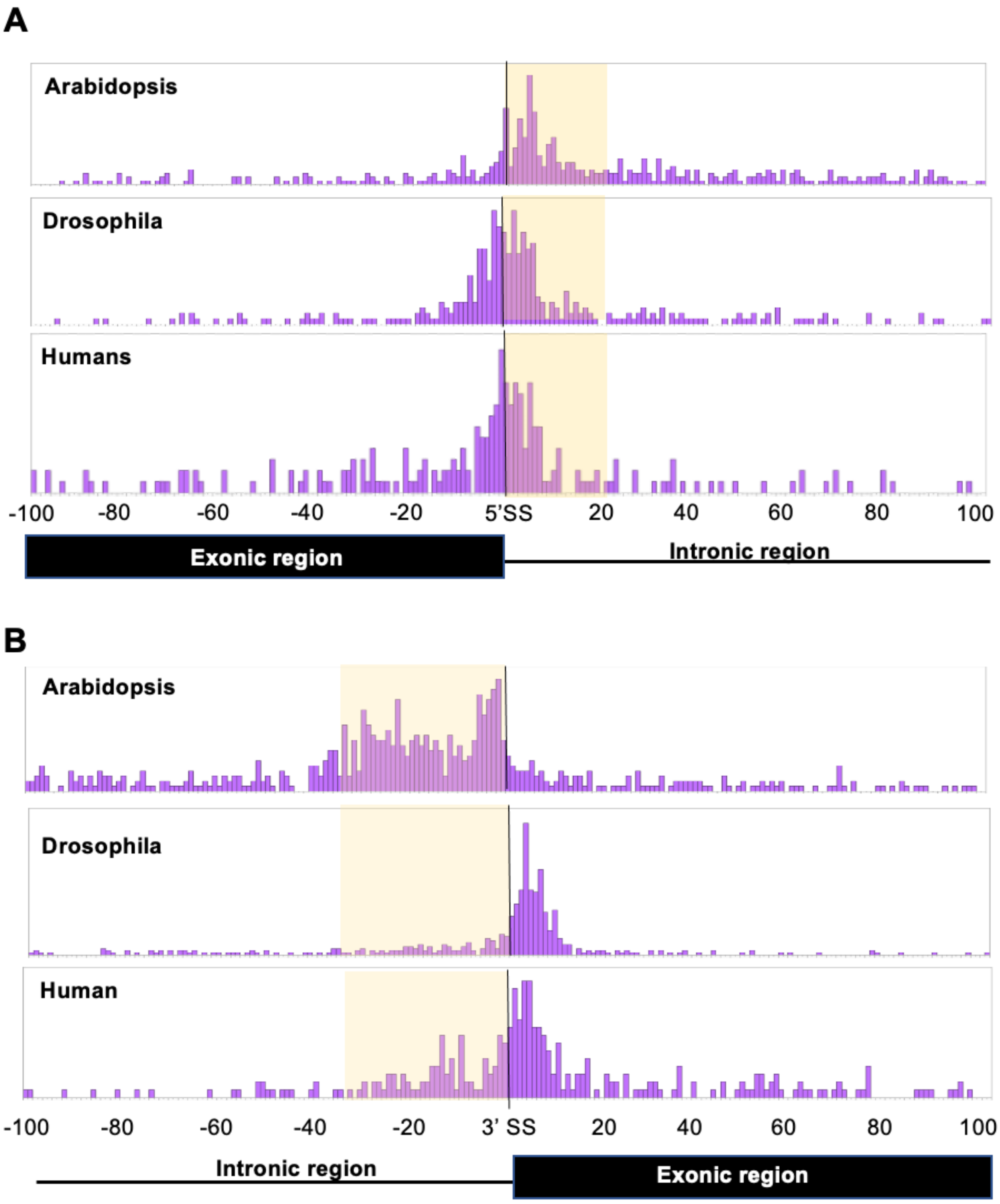

Supplementary Figure 5. Genetic variation affecting splice-site choice is often near the splice-site. A-B) Distribution of the distances of the closest associated SNPs

detected in SpliSER-GWAS for donors (A) and acceptors (B) in Arabidopsis, Drosophila and Humans. SS indicates the splice site. Intronic regions with higher number of associations is shaded for clarity.

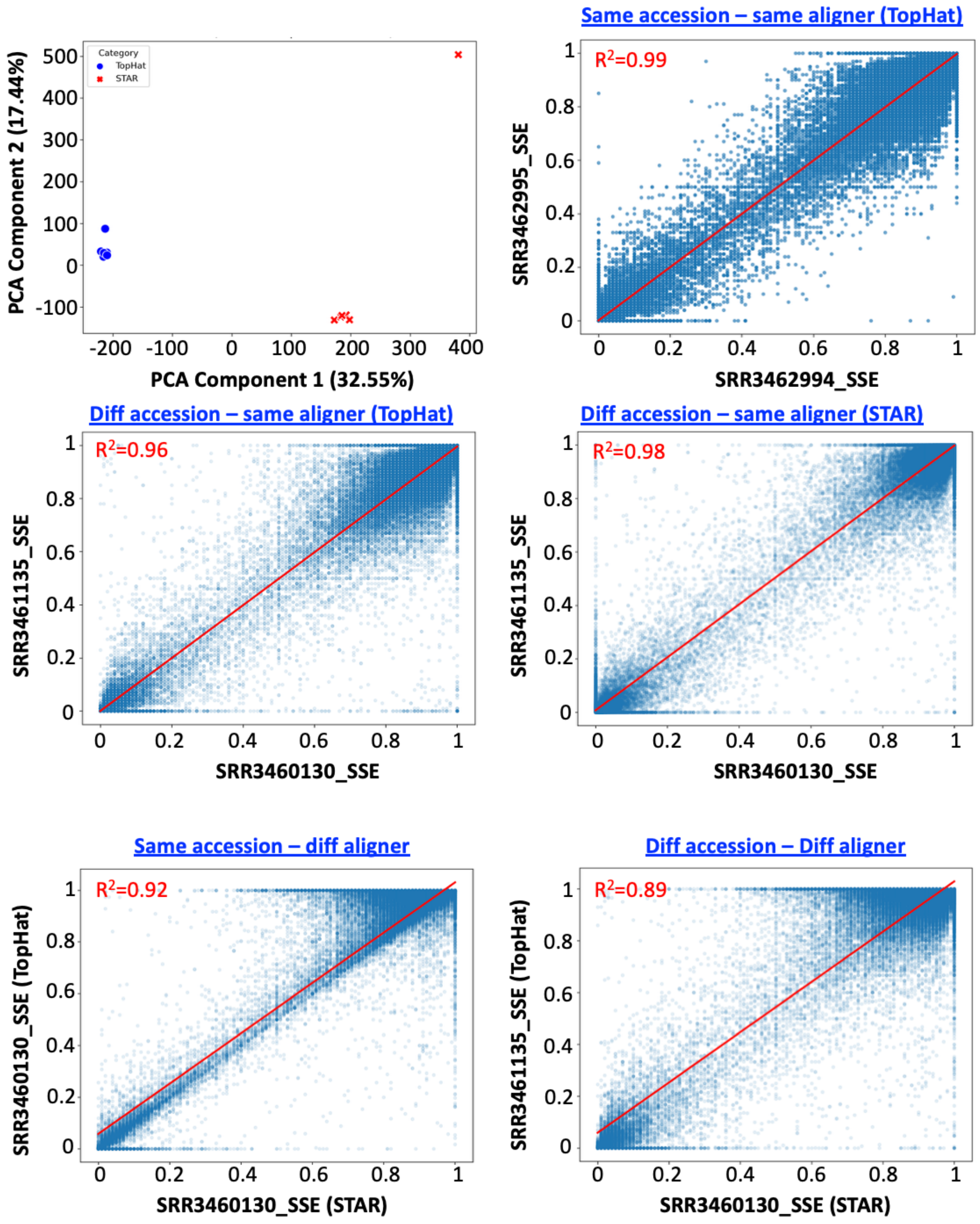

Supplementary Figure 6. Different aligners are unlikely to cause differences in genetic mapping of splicing variation. PCA analysis of splice-site strength of 7 different

accessions aligned with TopHat or STAR shows that accessions group based on the aligner, which suggests that as long as the aligner is the same within the species, it is unlikely to be causing an impact on mapping genetic variation in splicing. Correlation plots of the SSEs between different combinations of aligners and accessions show relatively minor impacts on correlation. The SRR numbers refer to the unique accession IDs of the samples used in the analysis.

Donors

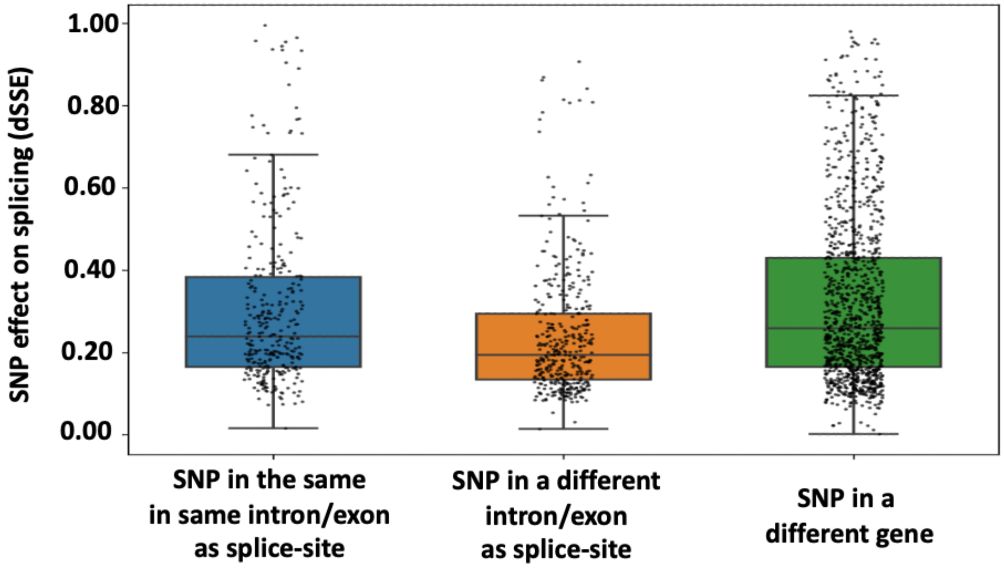

Acceptors

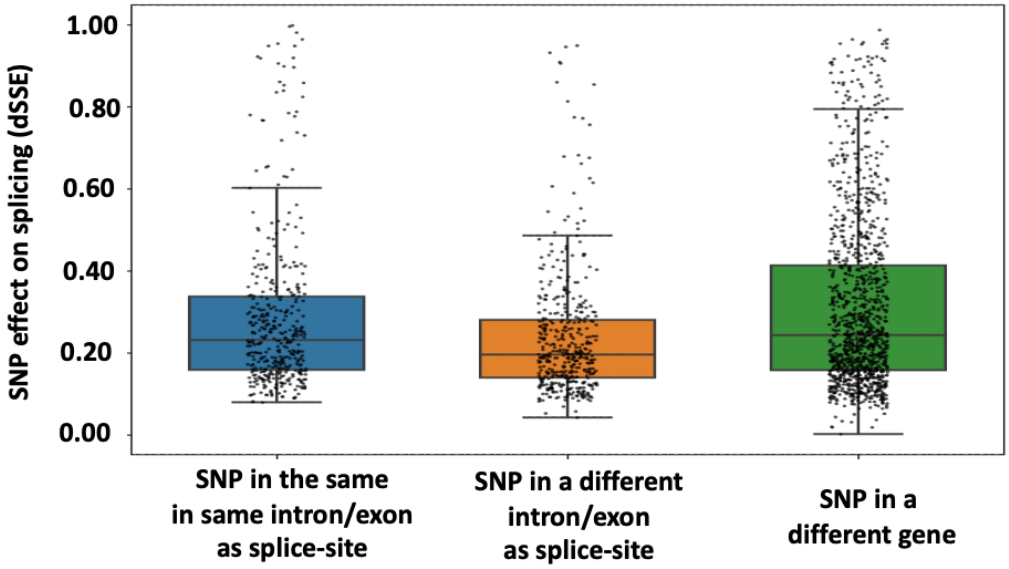

Supplementary Figure 7. Genetic variation proximal to the splice site had a stronger effect. Differences in the splice-site strengths of the two alleles (dSSE) is plotted on the basis of the occurrence of the SNP position.

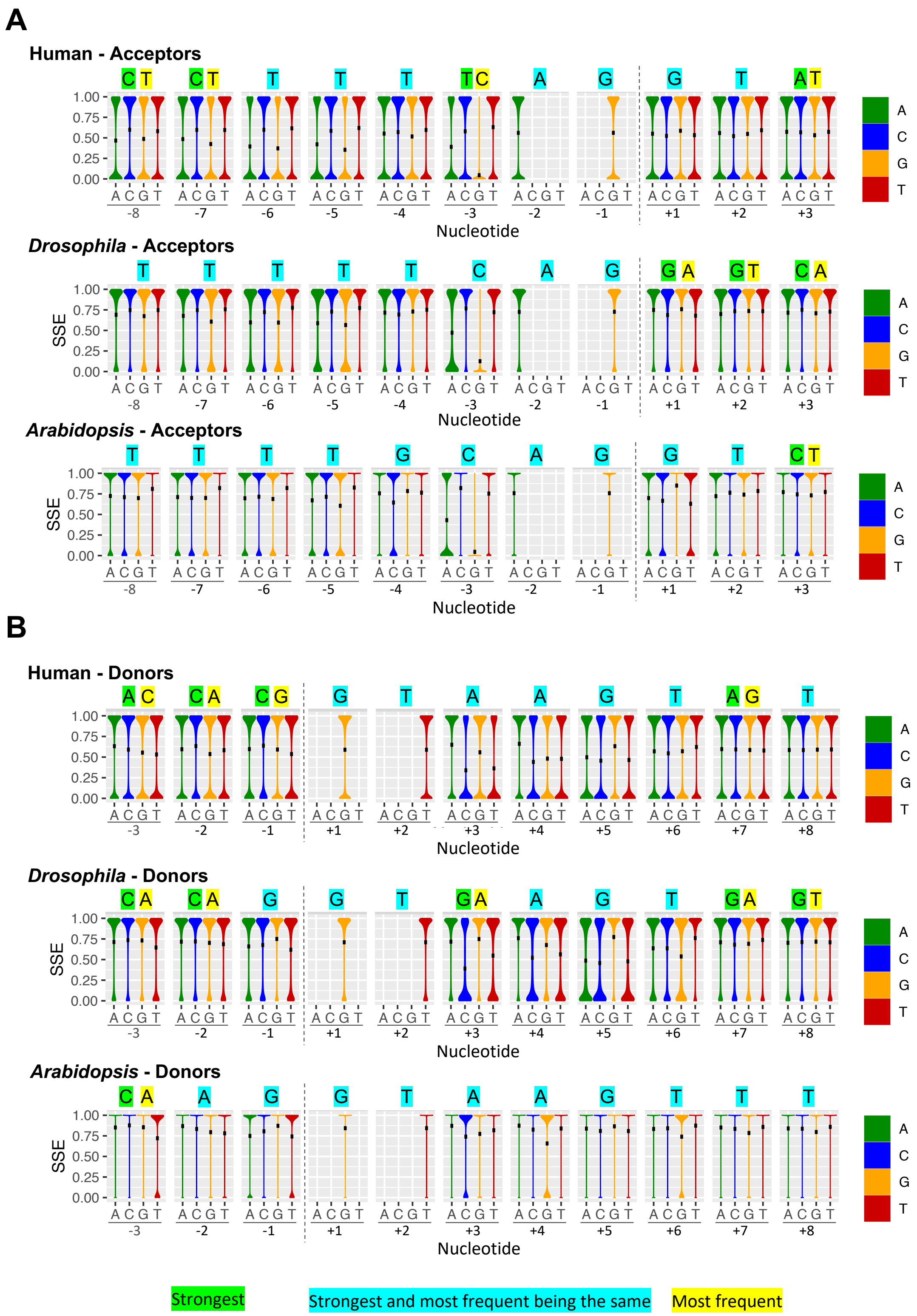

Supplementary Figure 8. The mean Splice-site Strength Estimate (SSE) of splice sites harbouring each of the four possible nucleotides at each position around the

splice site. A] Splice acceptor sites (AG only) from position -8 to +3. B] Splice donor sites (GT only) from position -3 to +8. Black dots represent the mean. Downsampled in each species to 1-2 million site/sequence/strength combinations. The nucleotides that are the strongest in terms of splice-site strength and also the most abundant are shown in cyan. If these two differ then both are shown side by side with the strongest in green and the most frequent in yellow.

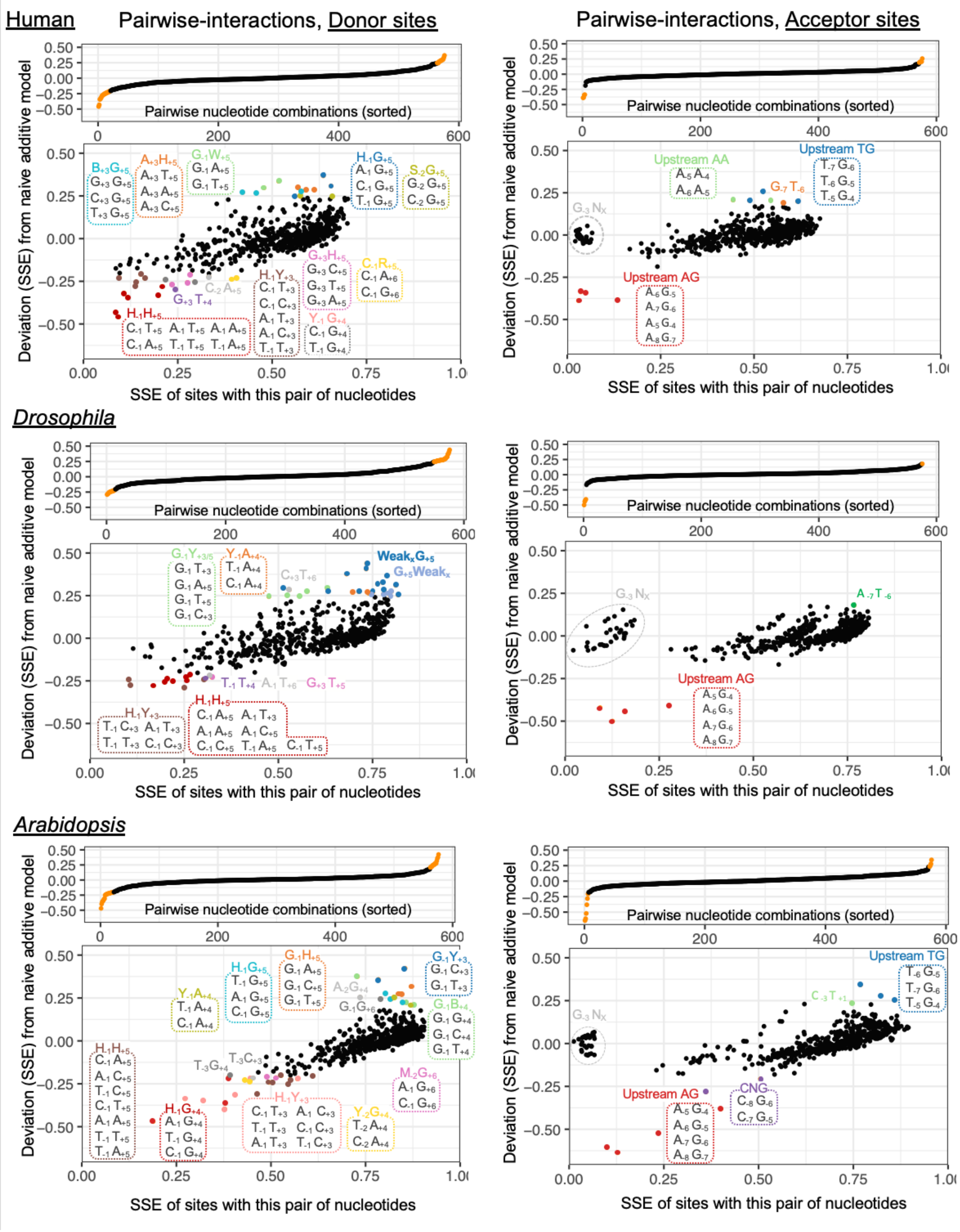

Human
Pairwise-interactions, Donor sites
Pairwise-interactions, Acceptor sites
Drosophila
Arabidopsis
Deviation (SSE) from naive additive model
Pairwise nucleotide combinations (sorted)
SSE of sites with this pair of nucleotides
Upstream AA
Upstream TG
Upstream AG
CNG

Supplementary Figure 9. Pairwise combinations of nucleotides around the splice site whose Splice-site Strength Estimate (SSE) deviate from a naïve additive model for human (top), Drosophila (middle) and Arabidopsis (bottom). Left – Donor sites (GT only), Right - Acceptor sites (AG only). Each point represents a single pairwise combination of nucleotides around the splice site, -3 to +8 in donors, and -8 to +3 in acceptors. Upper panels – nucleotide pairs which deviate from the additive model are identified in orange. Lower panels – nucleotide pairs are plotted with their SSE (x-axis) and deviation from the additive model (y-axis) are identified in orange.

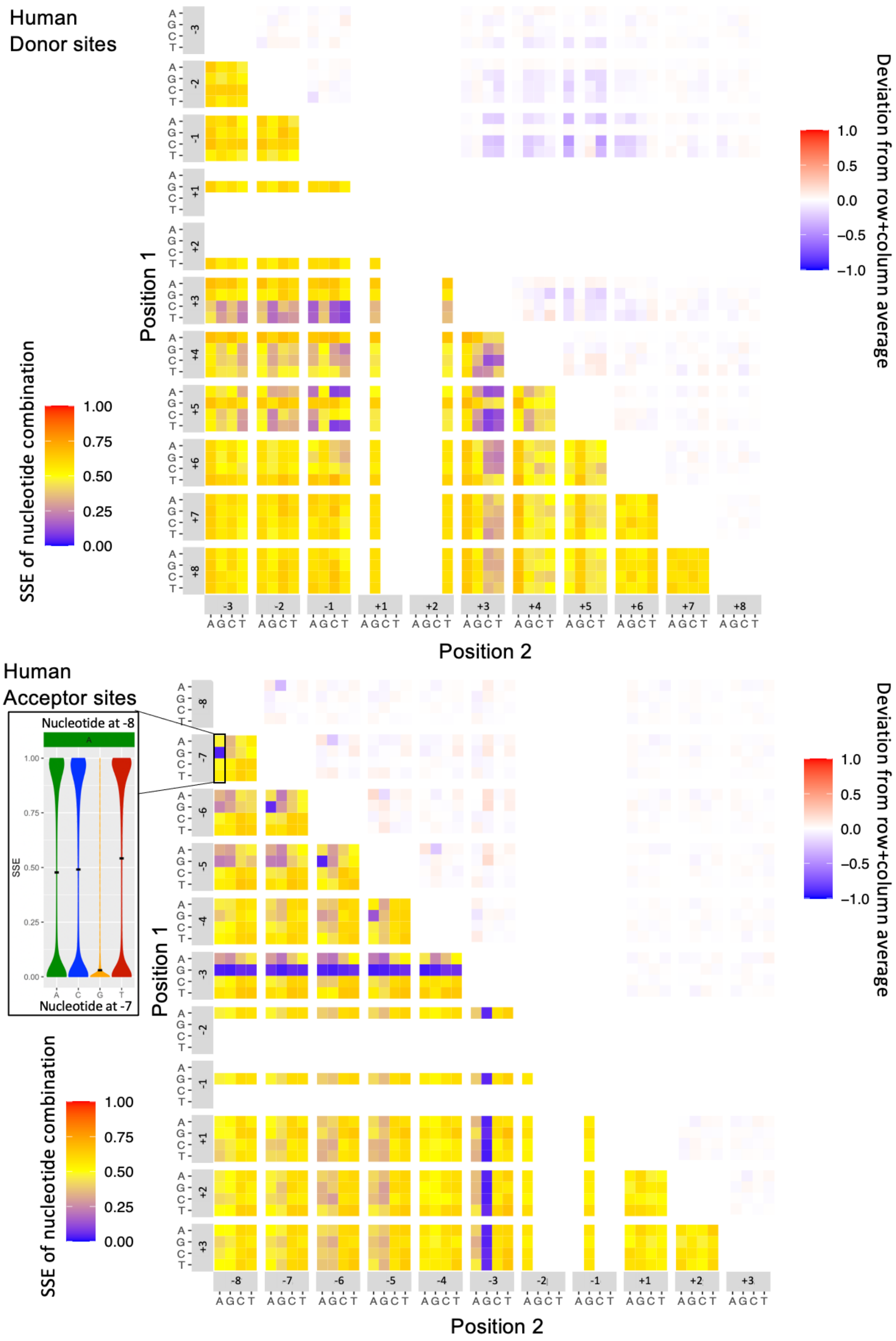
Human
Donor sites
SSE of nucleotide combination
1.00
0.75
0.50
0.25
0.00
Position 1
Position 2
Deviation from row+column average
1.0
0.5
0.0
−0.5
−1.0
-3
-2
-1
+1
+2
+3
+4
+5
+6
+7
+8
A G C T
Human
Acceptor sites
Nucleotide at -8
SSE
Nucleotide at -7
SSE of nucleotide combination
1.00
0.75
0.50
0.25
0.00
Position 1
Position 2
Deviation from row+column average
1.0
0.5
0.0
−0.5
−1.0
-8
-7
-6
-5
-4
-3
-2
-1
+1
+2
+3
A G C T

Supplementary Figure 10. Heatmaps of Splice-site Strength Estimates (SSE) of pairwise combinations of nucleotides around human splice sites. Top – Donor sites (GT only), Bottom – Acceptor sites (AG only). Bottom left of plots show the SSE of the combination. Top right of plots shows the deviation of these combinations from an additive model. Inset shows the SSE of nucleotides at the -7 position of splice acceptor sites which harbor an adenine at position -8.

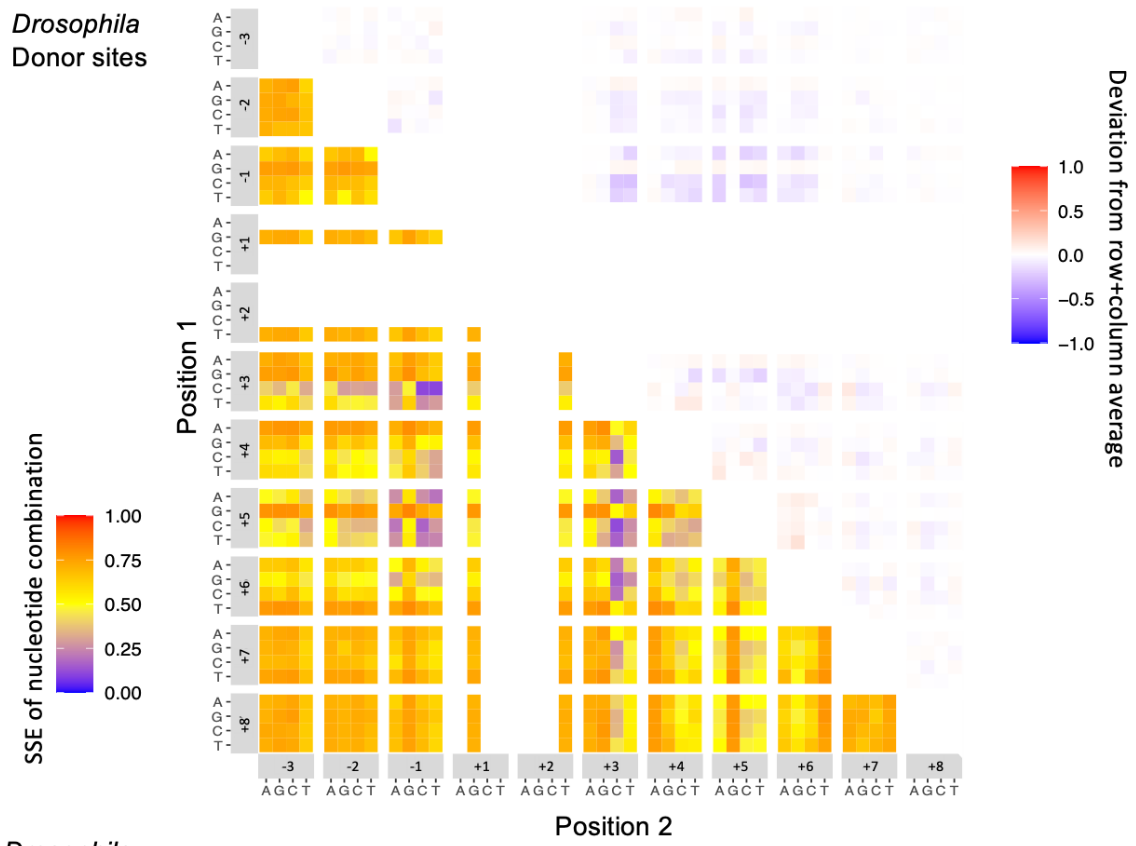
Drosophila
Donor sites
Position 1
Position 2
-3 -2 -1 +1 +2 +3 +4 +5 +6 +7 +8
A G C T
SSE of nucleotide combination
1.00
0.75
0.50
0.25
0.00
Deviation from row+column average
1.0
0.5
0.0
−0.5
−1.0

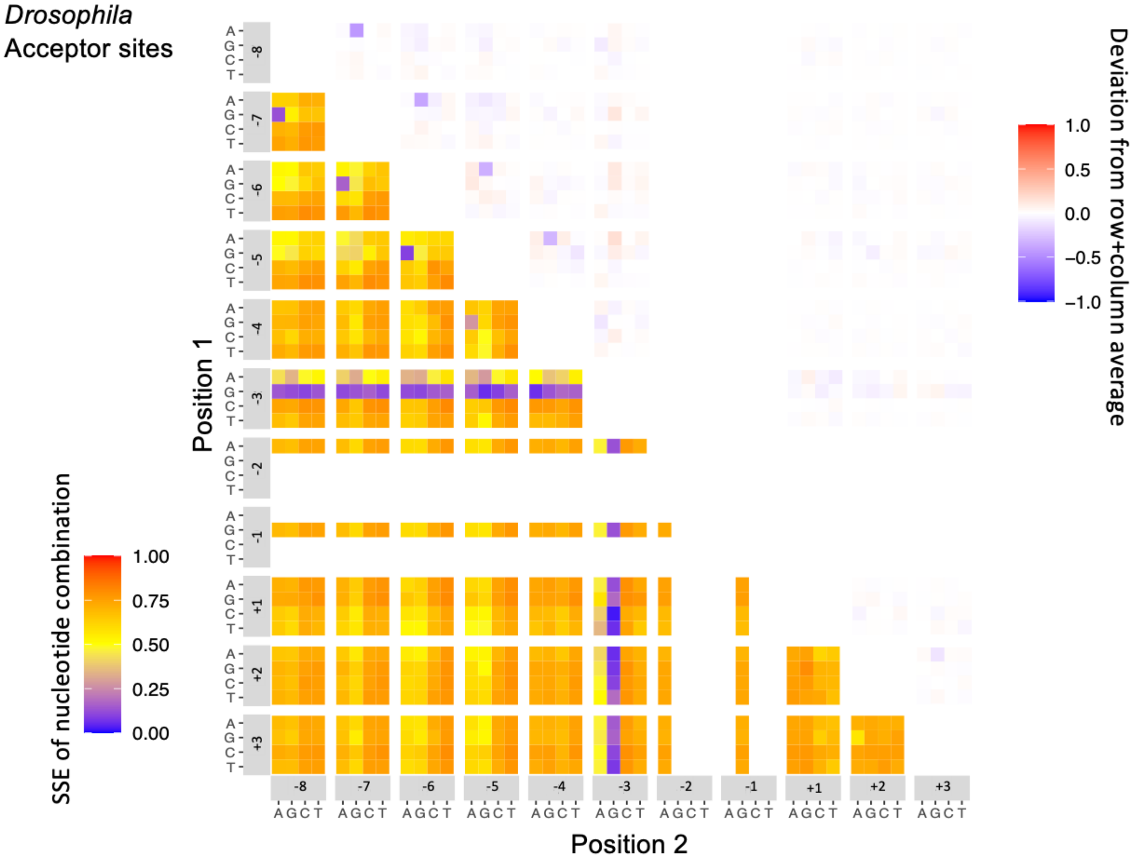
Drosophila
Acceptor sites
Position 1
Position 2
-8 -7 -6 -5 -4 -3 -2 -1 +1 +2 +3
A G C T
SSE of nucleotide combination
1.00
0.75
0.50
0.25
0.00
Deviation from row+column average
1.0
0.5
0.0
−0.5
−1.0

Supplementary Figure 11. Heatmaps of Splice-site Strength Estimates (SSE) of pairwise combinations of nucleotides around Drosophila splice sites. Top – Donor sites (GT only), Bottom – Acceptor sites (AG only). Bottom left of plots show the SSE of the combination. Top right of plots shows the deviation of these combinations from an additive model.

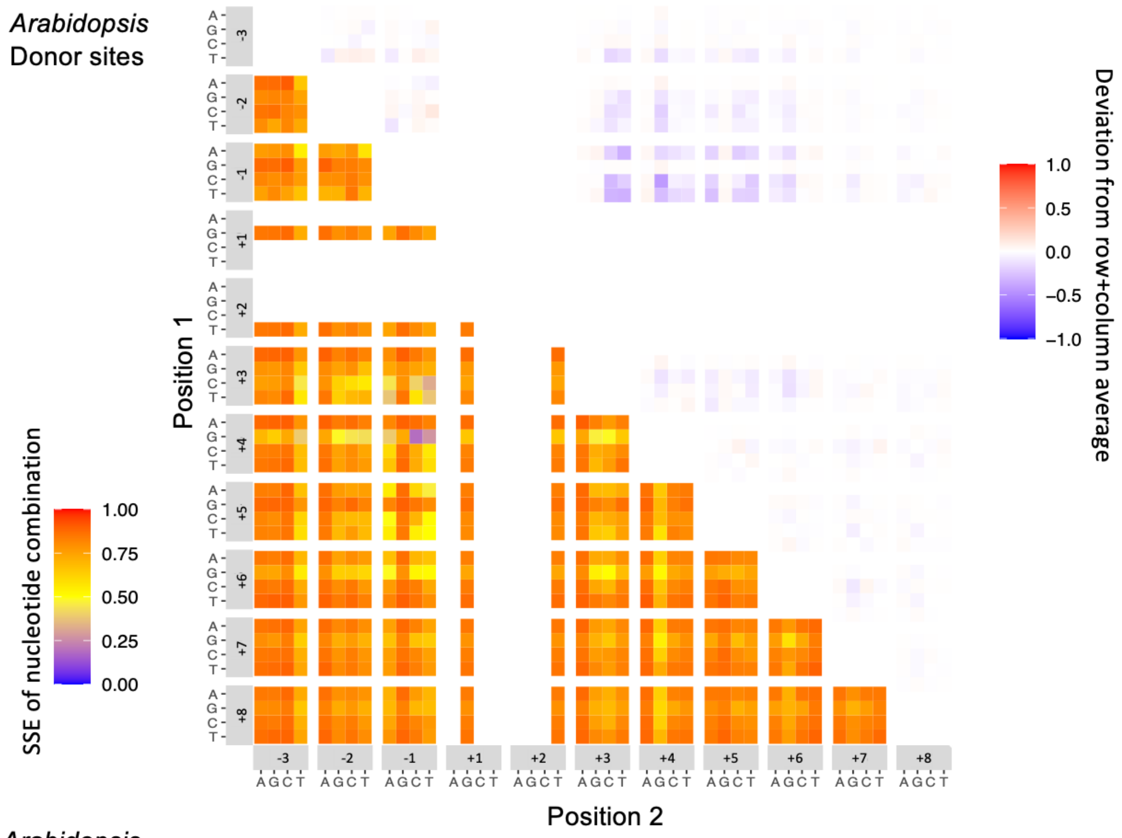
Arabidopsis
Donor sites
Position 1
Position 2
-3 -2 -1 +1 +2 +3 +4 +5 +6 +7 +8
A G C T
SSE of nucleotide combination
1.00
0.75
0.50
0.25
0.00
Deviation from row+column average
1.0
0.5
0.0
−0.5
−1.0

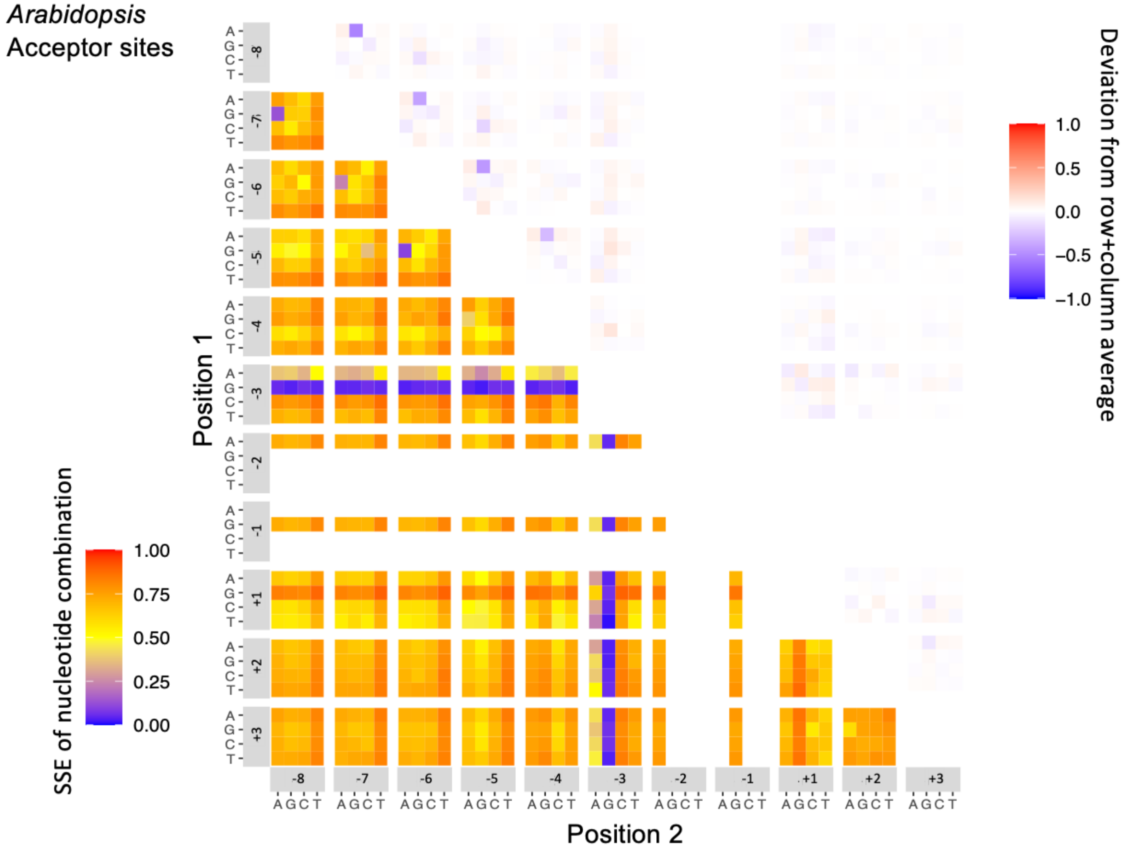
Arabidopsis
Acceptor sites
Position 1
Position 2
-8 -7 -6 -5 -4 -3 -2 -1 +1 +2 +3
A G C T
SSE of nucleotide combination
1.00
0.75
0.50
0.25
0.00
Deviation from row+column average
1.0
0.5
0.0
−0.5
−1.0

Supplementary Figure 12. Heatmaps of Splice-site Strength Estimates (SSE) of pairwise combinations of nucleotides around Arabidopsis splice sites. Top – Donor sites (GT only), Bottom – Acceptor sites (AG only). Bottom left of plots show the SSE of the combination. Top right of plots shows the deviation of these combinations from an additive model.

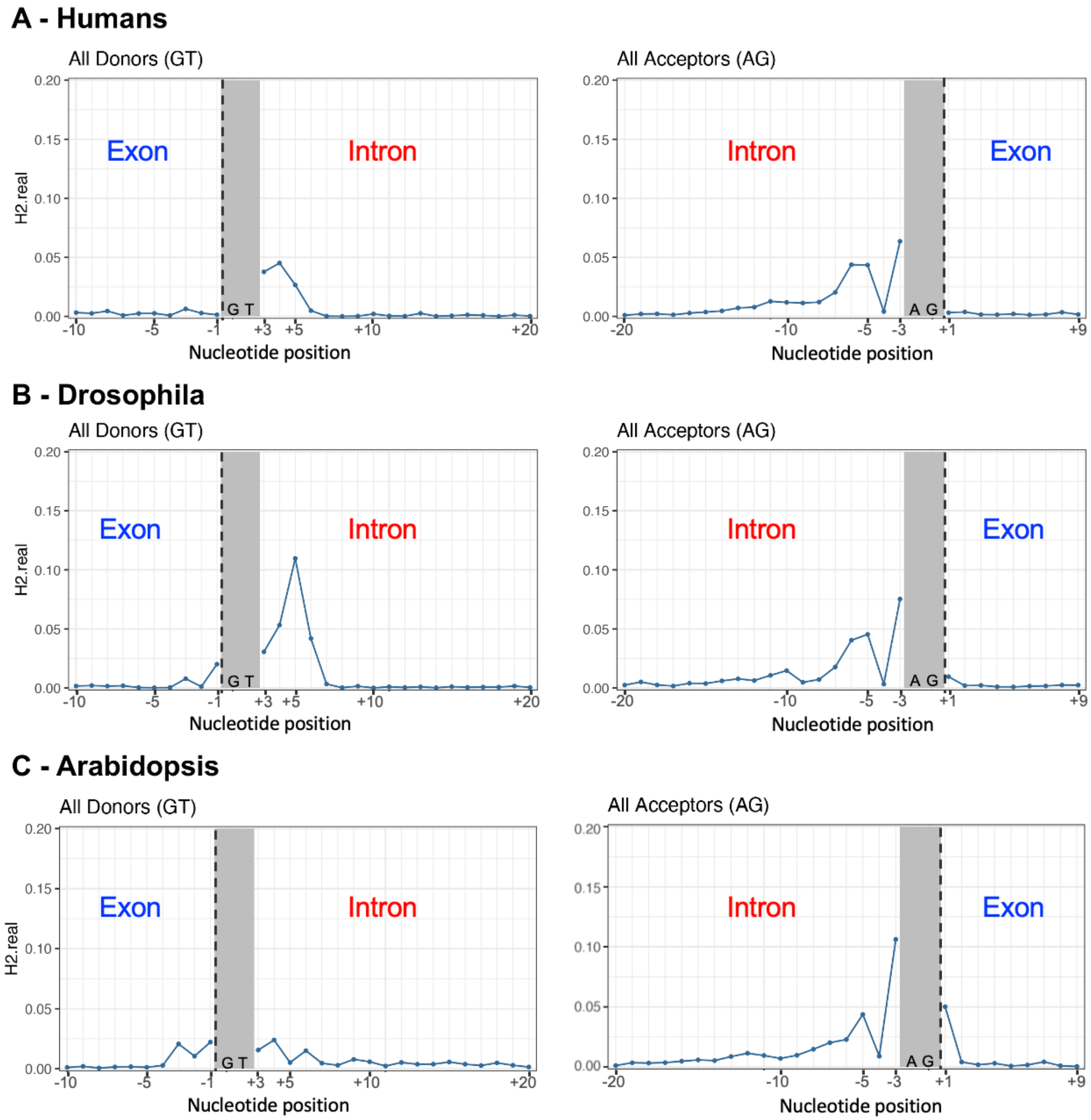

Supplementary Figure 13. Intronic variability is the primary driver of variation in splice-site strength. Realized heritability for every single nucleotide position surrounding splice-sites in Humans (A), Drosophila (B) and Arabidopsis (C). The grey region represents GT or AG, which do not have any polymorphisms, since the analysis is restricted to splice-sites harboring the consensus GT or AG sequences. The intronic

regions explain more variability in splice-site usage than the exonic regions in all three species.

A

Splice Promoting — **TS=CS ONLY**

Donors

	-2	-1	+1	+2	+3	+4	+5	+6
G	6	34	22	2	7	3	39	5
A	14	4	5	0	29	24	2	8
T	8	3	3	23	5	2	7	11
C	6	2	2	6	1	1	5	5
Total	34	43	32	31	42	30	53	29
Best	A	G	G	T	A	A	G	T
	41%	79%	69%	74%	69%	80%	74%	38%

Acceptors

	-6	-5	-4	-3	-2	-1	+1	+2
G	6	3	8	1	2	26	19	6
A	3	3	7	7	19	5	5	11
T	11	12	2	9	4	3	10	16
C	5	7	7	22	0	1	8	12
Total	25	25	24	39	25	35	42	45
Best	T	T	G	C	A	G	G	T
	44%	48%	33%	56%	76%	74%	45%	35%

Splice Reducing — **TS=CS ONLY**

Donors

	-2	-1	+1	+2	+3	+4	+5	+6
G	12	5	6	2	14	6	3	9
A	4	21	14	3	6	4	28	5
T	7	12	8	8	11	11	17	5
C	11	5	4	18	11	9	5	10
Total	34	43	32	31	42	30	53	29
Best	G	A	A	C	G	T	A	C
	35%	49%	44%	58%	33%	36%	53%	34%

Acceptors

	-6	-5	-4	-3	-2	-1	+1	+2
G	8	6	6	8	10	6	2	16
A	8	5	8	11	6	18	19	11
T	2	4	3	12	7	5	12	6
C	7	10	7	8	2	6	9	12
Total	25	25	24	39	25	35	42	75
Best	G/A	C	A	T	G	A	A	G
	32%	40%	33%	31%	40%	51%	45%	21%

B

Splice Promoting — **TS ONLY**

Donors

	-2	-1	+1	+2	+3	+4	+5	+6
G	6	34	23	2	7	3	39	4
A	15	4	5	1	33	26	2	9
T	8	3	3	24	6	3	8	14
C	6	2	2	6	1	1	5	5
Total	35	43	33	33	47	33	54	32
Best	A	G	G	T	A	A	G	T
	41%	79%	69%	74%	69%	80%	74%	38%

Acceptors

	-6	-5	-4	-3	-2	-1	+1	+2
G	8	3	9	1	3	26	19	6
A	3	4	8	7	19	5	5	11
T	13	14	2	10	4	3	10	18
C	6	8	7	22	0	1	8	12
Total	30	29	26	40	26	35	42	47
Best	T	T	G	C	A	G	G	T
	44%	48%	35%	55%	73%	74%	45%	38%

Splice Reducing — **TS ONLY**

Donors

	-2	-1	+1	+2	+3	+4	+5	+6
G	13	5	6	3	17	7	3	10
A	4	21	15	3	6	4	28	6
T	7	12	8	8	12	11	16	5
C	11	5	4	19	12	11	7	11
Total	35	43	33	33	47	33	54	32
Best	G	A	A	C	G	T/C	A	C
	37%	49%	45%	58%	36%	33%	52%	34%

Acceptors

	-6	-5	-4	-3	-2	-1	+1	+2
G	9	7	6	8	10	6	2	17
A	11	6	9	11	6	18	19	11
T	2	5	3	12	8	5	12	6
C	8	11	8	9	2	6	9	13
Total	30	29	26	40	26	35	42	47
Best	A	C	A	T	G	A	A	G
	37%	38%	35%	31%	38%	51%	45%	36%

C

Splice Promoting — **CS ONLY**

Donors

	-2	-1	+1	+2	+3	+4	+5	+6
G	13	49	31	4	17	9	52	6
A	26	7	6	1	51	37	7	13
T	16	5	4	36	9	8	12	24
C	14	6	2	10	2	8	6	13
Total	69	67	43	51	79	62	77	56
Best	A	G	G	T	A	A	G	T
	38%	73%	72%	71%	65%	60%	68%	43%

Acceptors

	-6	-5	-4	-3	-2	-1	+1	+2
G	7	7	18	2	2	43	29	14
A	8	7	22	13	27	8	13	16
T	23	25	21	27	6	4	17	26
C	11	16	9	52	1	5	13	20
Total	49	55	70	94	36	60	72	76
Best	T	T	A	C	A	G	G	T
	47%	45%	31%	55%	75%	72%	40%	34%

Splice Reducing — **CS ONLY**

Donors

	-2	-1	+1	+2	+3	+4	+5	+6
G	22	6	7	5	34	16	7	14
A	11	32	19	7	11	10	37	13
T	19	19	12	12	18	17	23	10
C	17	10	5	27	16	19	10	19
Total	69	67	43	51	79	62	77	56
Best	G	A	A	C	G	C	A	C
	32%	48%	44%	53%	43%	31%	48%	34%

Acceptors

	-6	-5	-4	-3	-2	-1	+1	+2
G	15	11	22	12	14	9	8	23
A	10	14	20	22	9	33	29	20
T	9	13	14	41	10	9	16	12
C	15	17	14	19	3	9	19	21
Total	49	55	70	94	36	60	72	76
Best	G/C	C	G	T	G	A	A	G
	31%	31%	31%	44%	39%	55%	40%	30%

Supplementary Figure 14. High resolution SpliSER-GWAS allows inferring best nucleotides that promote splicing. The distribution of splice-promoting allelic variation for positions -2 to +6 around the splice-donor site and -6 to +2 around the splice-acceptor site based on associations from all three species considering either Top SNP (TS) (B), the closest SNP in the peak (CS) (C), or where both the CS and TS are the same (A). The most frequent splice-promoting nucleotide is highlighted for each position. For control to account for general sequence variation, splice-reducing nucleotides are also shown.

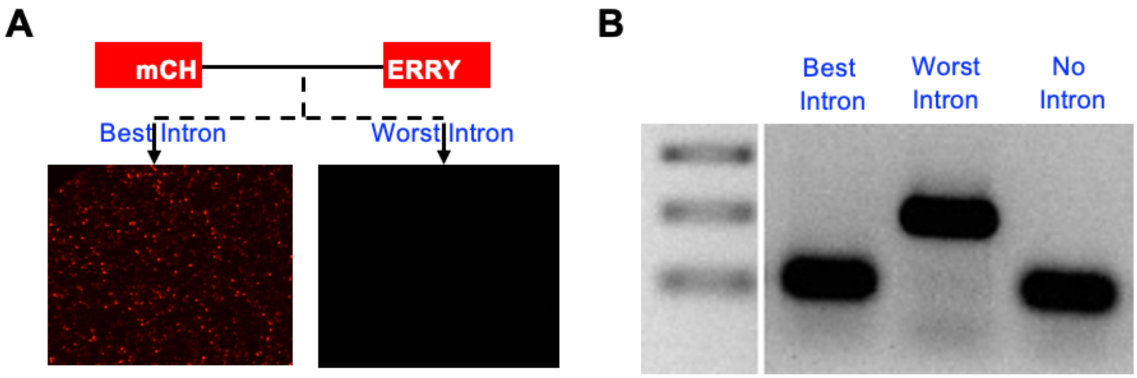

Supplementary Figure 15. Experimental verification of the performance of synthetic introns designed from the best and worst nucleotide combinations through an mCHERRY mini gene assay. Effect was assayed through mCHERRY fluorescence (A) and via RT-PCR (B).

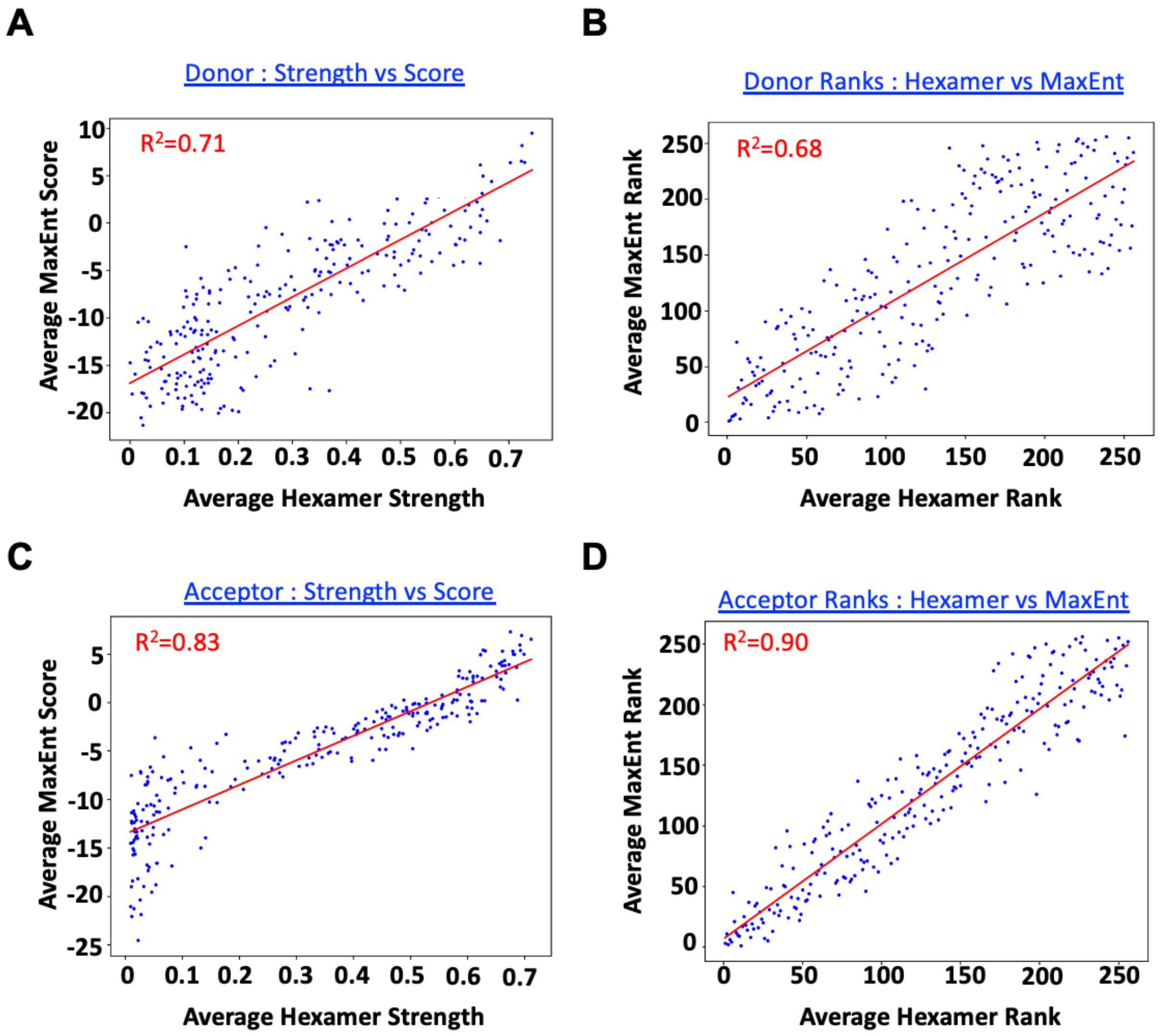

Supplementary Figure 16. Most of the variation in MaxEnt scores could be attributed to hexamer groupings. Correlation of the average hexamer strength with MaxEnt scores (A, C) and their rank correlation (B, D) for splice donors (A, B) and splice acceptors (C, D).

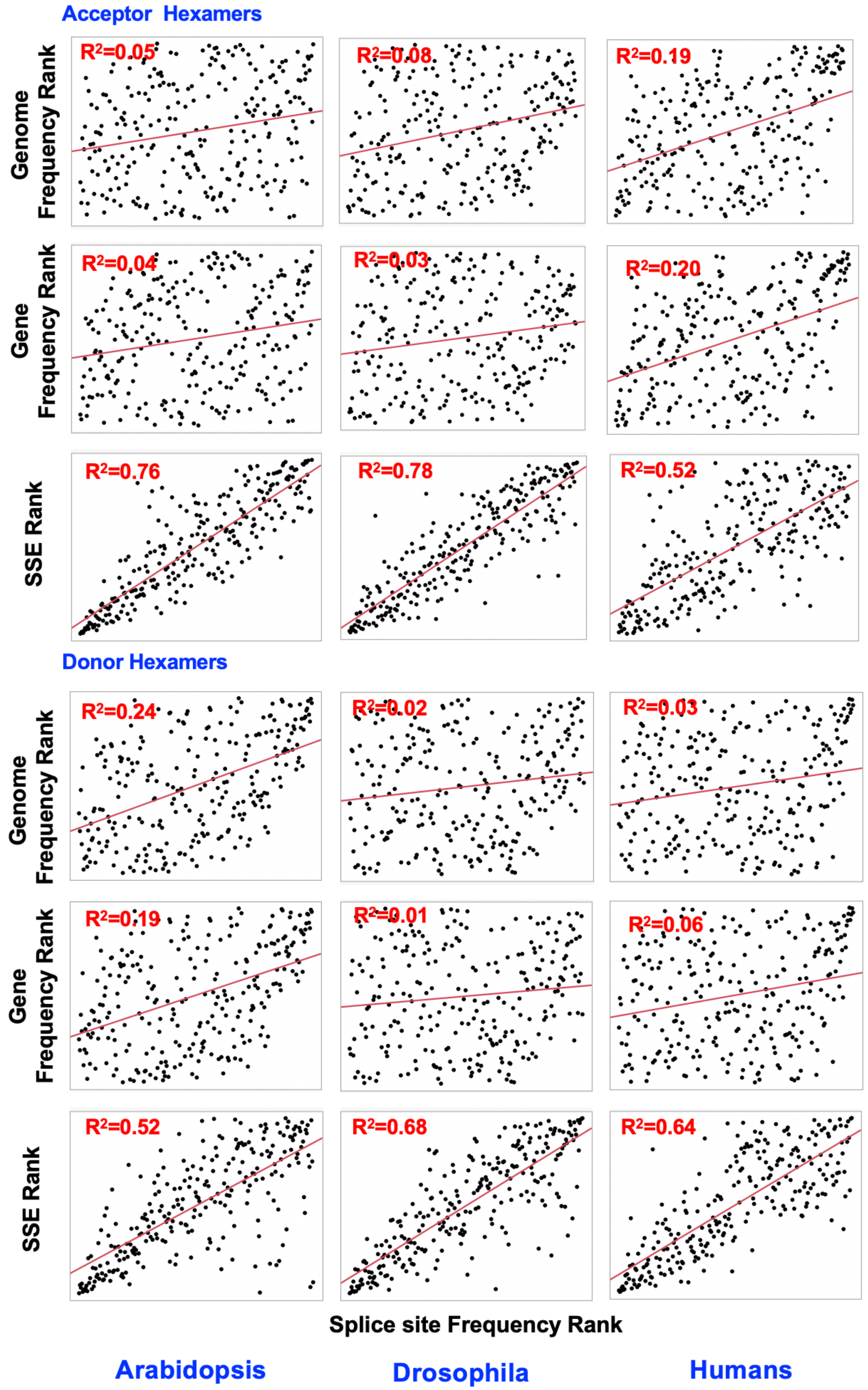
Acceptor Hexamers
Genome Frequency Rank
Gene Frequency Rank
SSE Rank
R²=0.05
R²=0.08
R²=0.19
R²=0.04
R²=0.03
R²=0.20
R²=0.76
R²=0.78
R²=0.52
Donor Hexamers
R²=0.24
R²=0.02
R²=0.03
R²=0.19
R²=0.01
R²=0.06
R²=0.52
R²=0.68
R²=0.64
Splice site Frequency Rank
Arabidopsis
Drosophila
Humans

Supplementary Figure 17. Hexamer frequency at the splice-sites is correlated with splice-site strength but not with the frequency of the hexamers in the genome or within protein coding genes. Hexamer frequency ranks are plotted against their corresponding frequency ranks in the genome, genes and the hexamer rankings based on splice-site strength estimates.

A

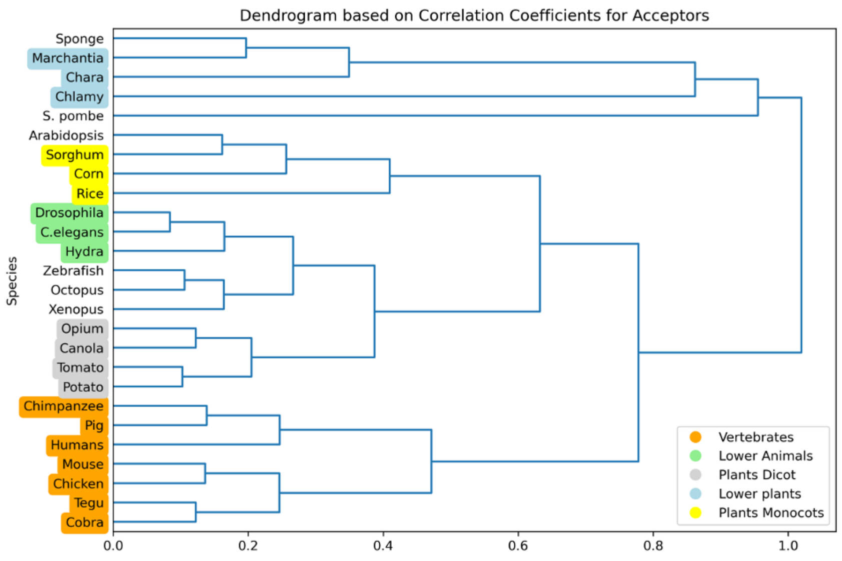

B

Average SSE - Donors

Hexamer Groups	p-Distance	Human	Arabidopsis	Drosophila
Group 1	0	0.742	0.940	0.843
Group 2	0.167	0.637	0.872	0.705
Group 3	0.333	0.472	0.796	0.549
Group 4	0.5	0.296	0.705	0.320
Group 5	0.667	0.129	0.560	0.170

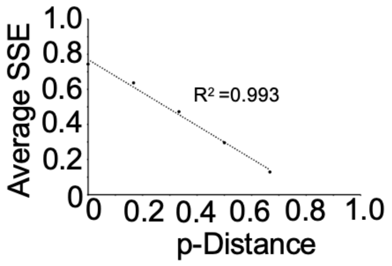

C

Average SSE - Acceptors

Hexamer Groups	p-Distance	Human	Arabidopsis	Drosophila
Group 1	0	0.691	0.907	0.825
Group 2	0.167	0.532	0.751	0.688
Group 3	0.333	0.403	0.570	0.522
Group 4	0.5	0.324	0.418	0.414
Group 5	0.667	0.261	0.288	0.336

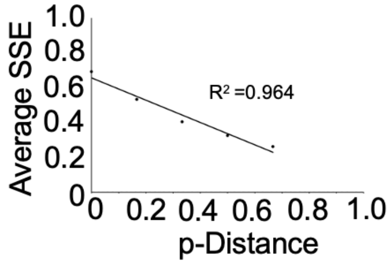

Supplementary Figure 18. Hexamer effect on donors could be accounted for by variation in U1 snRNA base pairing for donor sites (strongest hexamer for donors)

and sequence distance from the strongest hexamer for acceptors. A) A dendrogram of the rank correlations based on the hexamers surrounding acceptors in multiple species groups related species together. B-C) Sequence distance-based grouping of hexamers and their average strengths for donors (B) and acceptors (c) exhibits a near perfect correlation in all three species. Only the R^2 values for human are shown and other species displayed similar high correlations.

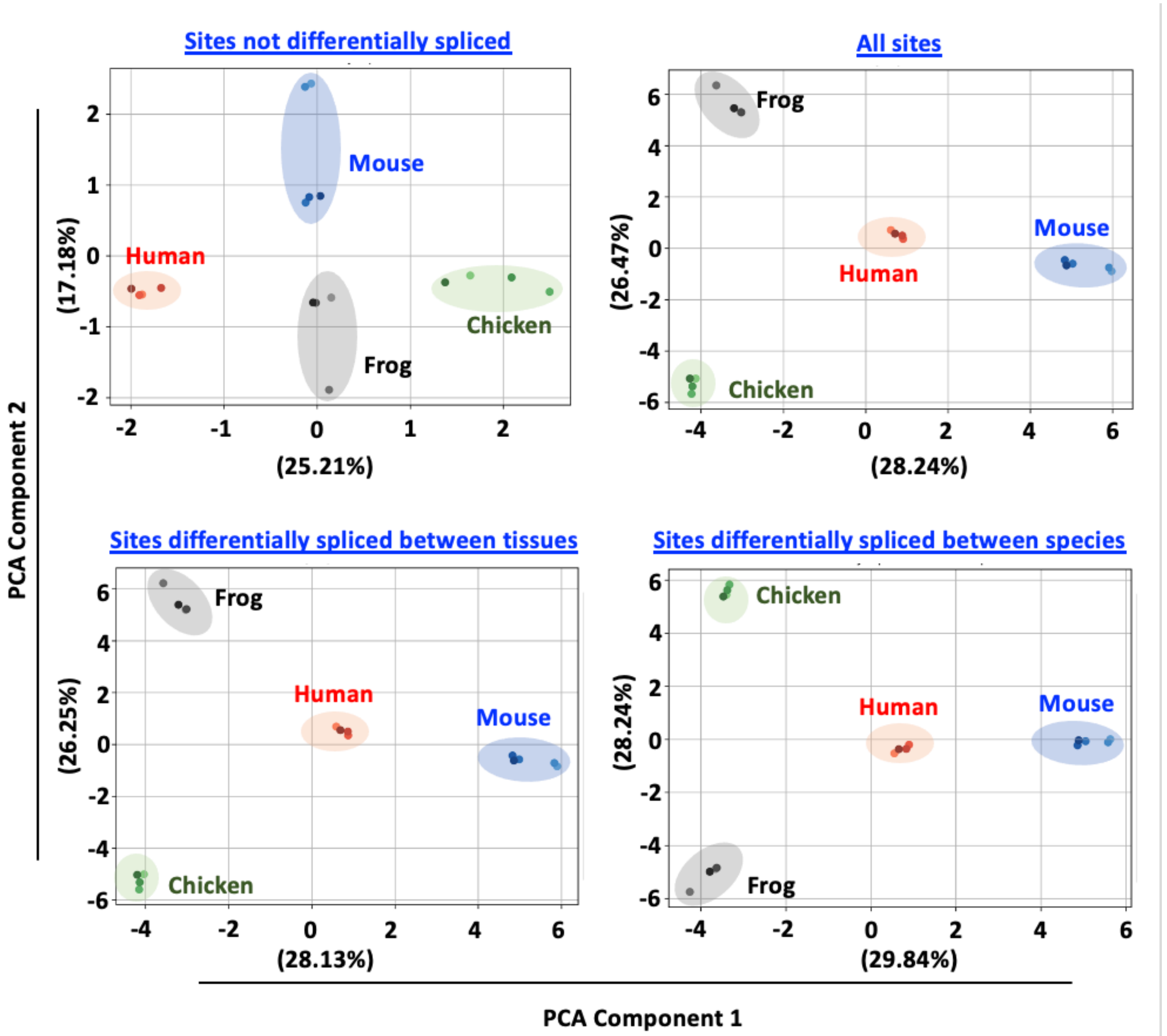

Supplementary Figure 19. Splicing is regulated more at the species level than at a tissue-level. PCA analysis of the SSEs of orthologous splice-sites from four different tissues (Heart, Brain, Muscle, Liver) of Human, Mouse, Chicken and Frog. Original data was from Barbosa-Morais et al {Barbosa-Morais, 2012 #58}. Data is shown for all sites, sites that are not differentially spliced, sites that are differentially spliced between species for the same tissue and sites that are differentially spliced between tissues for the same species. Data suggests that tissue-specific splicing is not the primary determinant that results in a PCA clustering of species together.

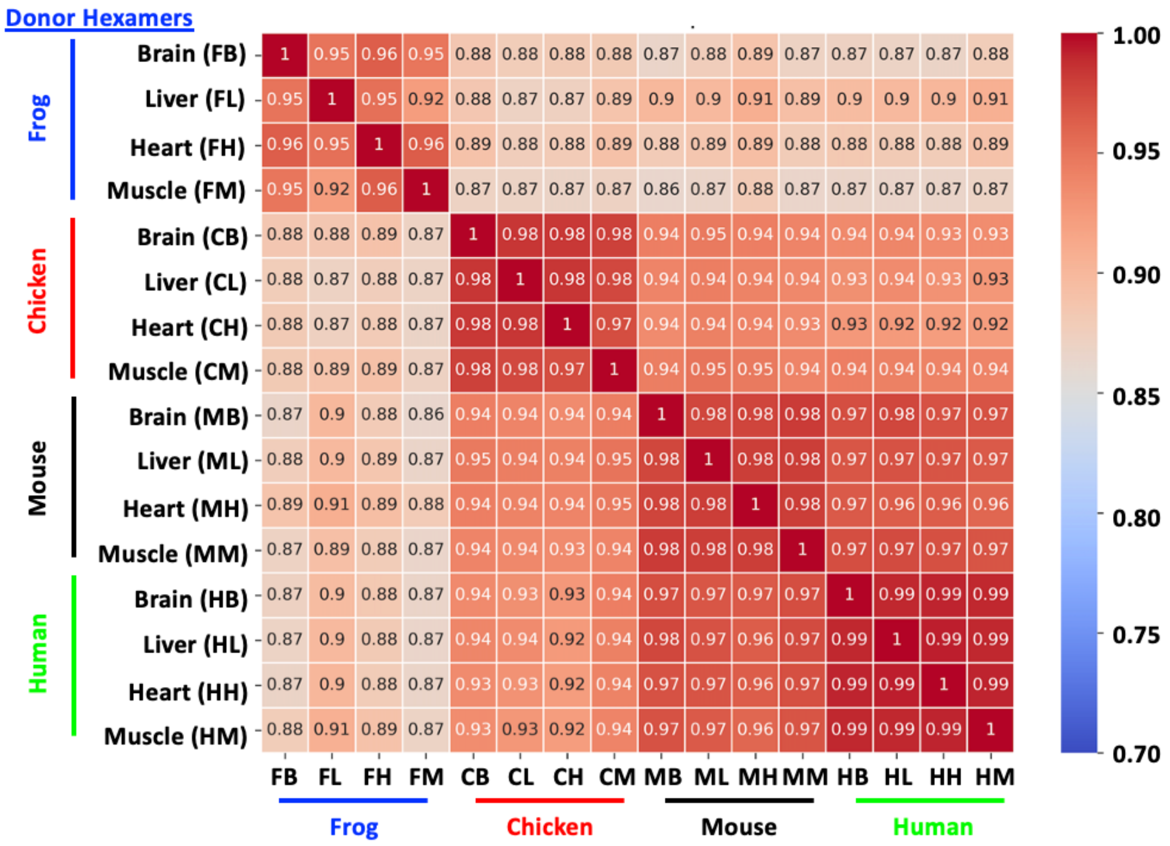
Donor Hexamers
Frog
Chicken
Mouse
Human
Brain (FB) 1 0.95 0.96 0.95 0.88 0.88 0.88 0.88 0.87 0.88 0.89 0.87 0.87 0.87 0.87 0.88
Liver (FL) 0.95 1 0.95 0.92 0.88 0.87 0.87 0.89 0.9 0.9 0.91 0.89 0.9 0.9 0.9 0.91
Heart (FH) 0.96 0.95 1 0.96 0.89 0.88 0.88 0.89 0.88 0.89 0.89 0.88 0.88 0.88 0.88 0.89
Muscle (FM) 0.95 0.92 0.96 1 0.87 0.87 0.87 0.87 0.86 0.87 0.88 0.87 0.87 0.87 0.87 0.87
Brain (CB) 0.88 0.88 0.89 0.87 1 0.98 0.98 0.98 0.94 0.95 0.94 0.94 0.94 0.94 0.93 0.93
Liver (CL) 0.88 0.87 0.88 0.87 0.98 1 0.98 0.98 0.94 0.94 0.94 0.94 0.93 0.94 0.93 0.93
Heart (CH) 0.88 0.87 0.88 0.87 0.98 0.98 1 0.97 0.94 0.94 0.94 0.93 0.93 0.92 0.92 0.92
Muscle (CM) 0.88 0.89 0.89 0.87 0.98 0.98 0.97 1 0.94 0.95 0.95 0.94 0.94 0.94 0.94 0.94
Brain (MB) 0.87 0.9 0.88 0.86 0.94 0.94 0.94 0.94 1 0.98 0.98 0.98 0.97 0.98 0.97 0.97
Liver (ML) 0.88 0.9 0.89 0.87 0.95 0.94 0.94 0.95 0.98 1 0.98 0.98 0.97 0.97 0.97 0.97
Heart (MH) 0.89 0.91 0.89 0.88 0.94 0.94 0.94 0.95 0.98 0.98 1 0.98 0.97 0.96 0.96 0.96
Muscle (MM) 0.87 0.89 0.88 0.87 0.94 0.94 0.93 0.94 0.98 0.98 0.98 1 0.97 0.97 0.97 0.97
Brain (HB) 0.87 0.9 0.88 0.87 0.94 0.93 0.93 0.94 0.97 0.97 0.97 0.97 1 0.99 0.99 0.99
Liver (HL) 0.87 0.9 0.88 0.87 0.94 0.94 0.92 0.94 0.98 0.97 0.96 0.97 0.99 1 0.99 0.99
Heart (HH) 0.87 0.9 0.88 0.87 0.93 0.93 0.92 0.94 0.97 0.97 0.96 0.97 0.99 0.99 1 0.99
Muscle (HM) 0.88 0.91 0.89 0.87 0.93 0.93 0.92 0.94 0.97 0.97 0.96 0.97 0.99 0.99 0.99 1
FB FL FH FM CB CL CH CM MB ML MH MM HB HL HH HM
Frog
Chicken
Mouse
Human
1.00
0.95
0.90
0.85
0.80
0.75
0.70

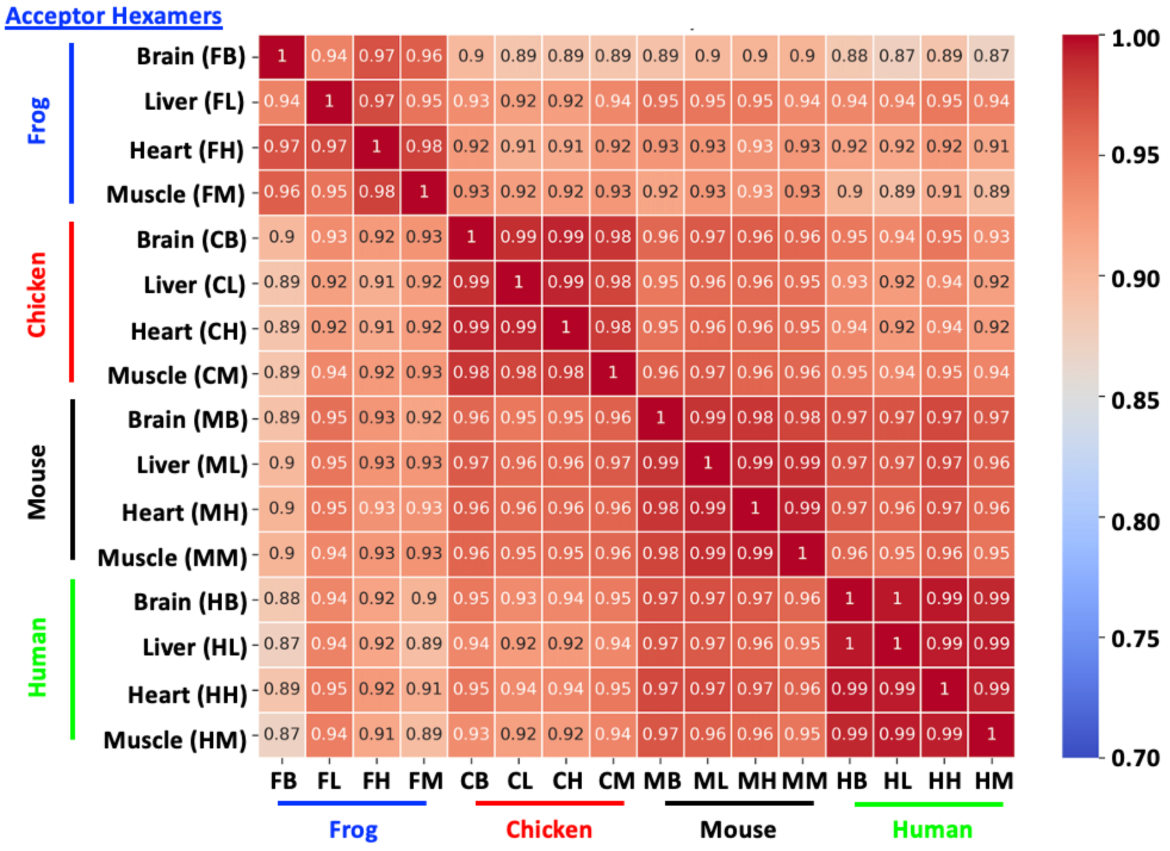
Acceptor Hexamers
Frog
Chicken
Mouse
Human
Brain (FB) 1 0.94 0.97 0.96 0.9 0.89 0.89 0.89 0.89 0.9 0.9 0.9 0.88 0.87 0.89 0.87
Liver (FL) 0.94 1 0.97 0.95 0.93 0.92 0.92 0.94 0.95 0.95 0.95 0.94 0.94 0.94 0.95 0.94
Heart (FH) 0.97 0.97 1 0.98 0.92 0.91 0.91 0.92 0.93 0.93 0.93 0.93 0.92 0.92 0.92 0.91
Muscle (FM) 0.96 0.95 0.98 1 0.93 0.92 0.92 0.93 0.92 0.93 0.93 0.93 0.9 0.89 0.91 0.89
Brain (CB) 0.9 0.93 0.92 0.93 1 0.99 0.99 0.98 0.96 0.97 0.96 0.96 0.95 0.94 0.95 0.93
Liver (CL) 0.89 0.92 0.91 0.92 0.99 1 0.99 0.98 0.95 0.96 0.96 0.95 0.93 0.92 0.94 0.92
Heart (CH) 0.89 0.92 0.91 0.92 0.99 0.99 1 0.98 0.95 0.96 0.96 0.95 0.94 0.92 0.94 0.92
Muscle (CM) 0.89 0.94 0.92 0.93 0.98 0.98 0.98 1 0.96 0.97 0.96 0.96 0.95 0.94 0.95 0.94
Brain (MB) 0.89 0.95 0.93 0.92 0.96 0.95 0.95 0.96 1 0.99 0.98 0.98 0.97 0.97 0.97 0.97
Liver (ML) 0.9 0.95 0.93 0.93 0.97 0.96 0.96 0.97 0.99 1 0.99 0.99 0.97 0.97 0.97 0.96
Heart (MH) 0.9 0.95 0.93 0.93 0.96 0.96 0.96 0.96 0.98 0.99 1 0.99 0.97 0.96 0.97 0.96
Muscle (MM) 0.9 0.94 0.93 0.93 0.96 0.95 0.95 0.96 0.98 0.99 0.99 1 0.96 0.95 0.96 0.95
Brain (HB) 0.88 0.94 0.92 0.9 0.95 0.93 0.94 0.95 0.97 0.97 0.97 0.96 1 1 0.99 0.99
Liver (HL) 0.87 0.94 0.92 0.89 0.94 0.92 0.92 0.94 0.97 0.97 0.96 0.95 1 1 0.99 0.99
Heart (HH) 0.89 0.95 0.92 0.91 0.95 0.94 0.94 0.95 0.97 0.97 0.97 0.96 0.99 0.99 1 0.99
Muscle (HM) 0.87 0.94 0.91 0.89 0.93 0.92 0.92 0.94 0.97 0.96 0.96 0.95 0.99 0.99 0.99 1
FB FL FH FM CB CL CH CM MB ML MH MM HB HL HH HM
Frog
Chicken
Mouse
Human
1.00
0.95
0.90
0.85
0.80
0.75
0.70

Supplementary Figure 20. Hexamer rankings correlate closely following species rather than tissue. Same data that was reported in Supplementary Fig 19 was used to make hexamer ranking correlations for splice donors and acceptors.

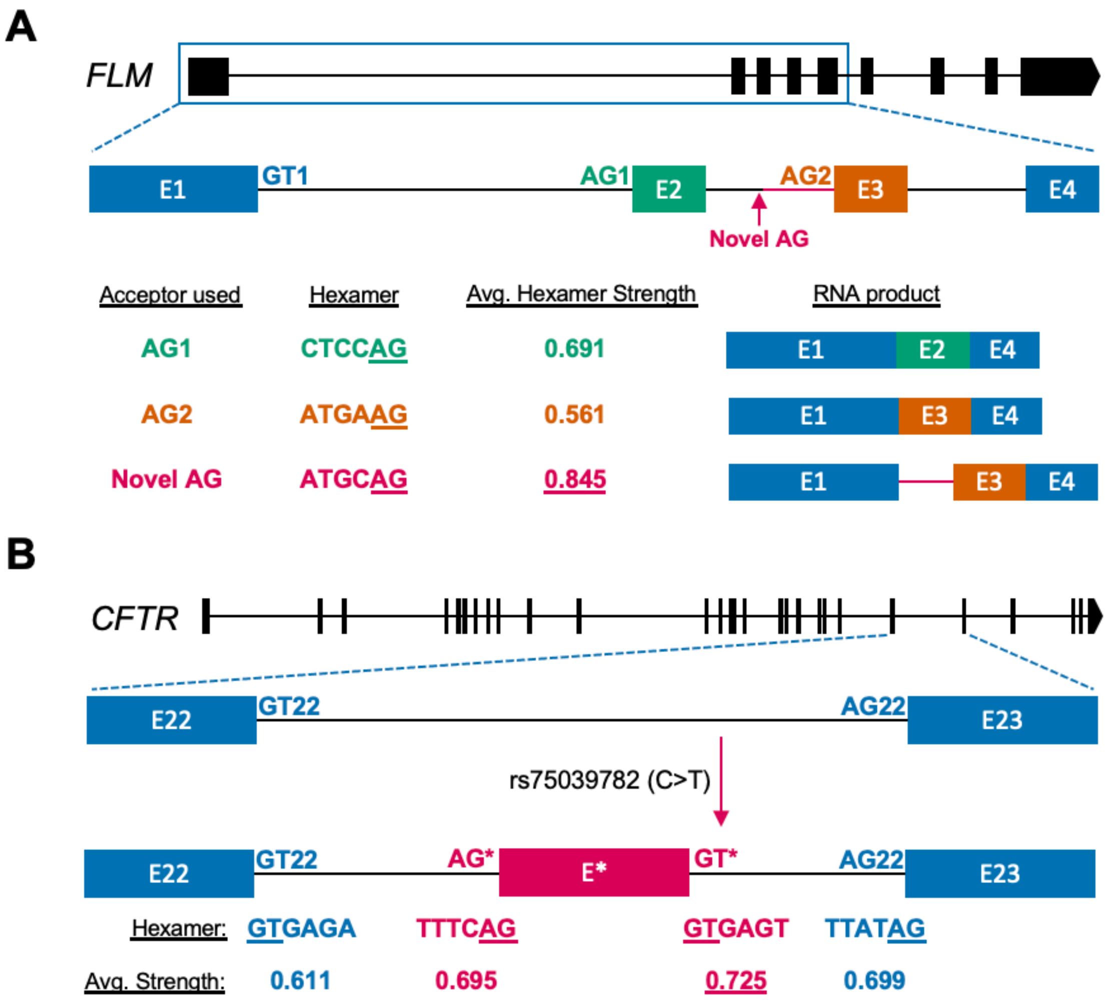

Supplementary Figure 21. Hexamer ranking explains mutational impacts on splice-site choice. A) A schematic representing the natural mutation that creates a competing AG at the *FLM* locus. RNA products arising out of the three competing AGs are shown along with their average splice-site strength. Hexamer ranking explains why AG1 is preferred over AG2 and why the new AG outcompetes both. B) Hexamer ranking explains why rs75039752 SNP at the CFTR gene leads to inclusion of a pseudo exon and leads to cystic fibrosis. rs75039752 introduces a new splice-site with the hexamer of higher rank that outcompetes the normal donor, which hijacks the natural partner. This allows another

acceptor, which is not normally used to be used now to include a pseudo exon, resulting in a frameshift and an impaired CFTR protein.

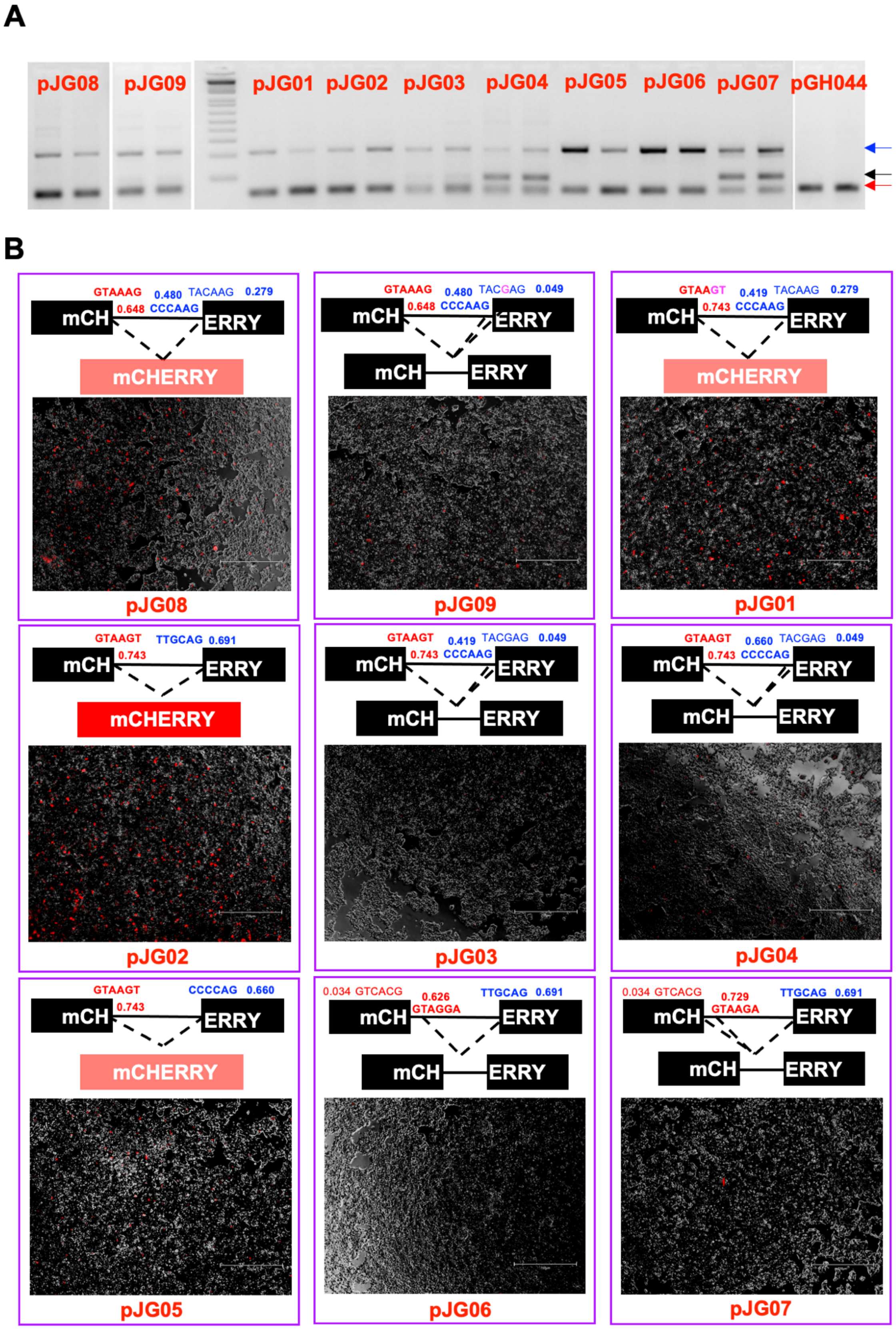
A
pJG08
pJG09
pJG01
pJG02
pJG03
pJG04
pJG05
pJG06
pJG07
pGH044
B
GTAAAG 0.480 TACAAG 0.279
0.648 CCCAAG
mCH
ERRY
mCHERRY
pJG08
GTAAAG 0.480 TACGAG 0.049
0.648 CCCAAG
mCH
ERRY
pJG09
GTAAGT 0.419 TACAAG 0.279
0.743 CCCAAG
mCHERRY
pJG01
GTAAGT TTGCAG 0.691
0.743
mCHERRY
pJG02
GTAAGT 0.419 TACGAG 0.049
0.743 CCCAAG
pJG03
GTAAGT 0.660 TACGAG 0.049
0.743 CCCCAG
pJG04
GTAAGT CCCCAG 0.660
0.743
mCHERRY
pJG05
0.034 GTCACG 0.626 TTGCAG 0.691
GTAGGA
pJG06
0.034 GTCACG 0.729 TTGCAG 0.691
GTAAGA
pJG07

Supplementary Figure 22. Hexamer variations confer differential splicing that is consistent with the hexamer rank order. A) RT-PCR analysis of splicing with different hexamer sequence variants in the *MYO15B* intron. B) mCHERRY mini gene assays with *MYO15B* intron with various hexamer combinations. The specific mutations are shown in purple, and the donor and acceptors are shown in red and blue, respectively.

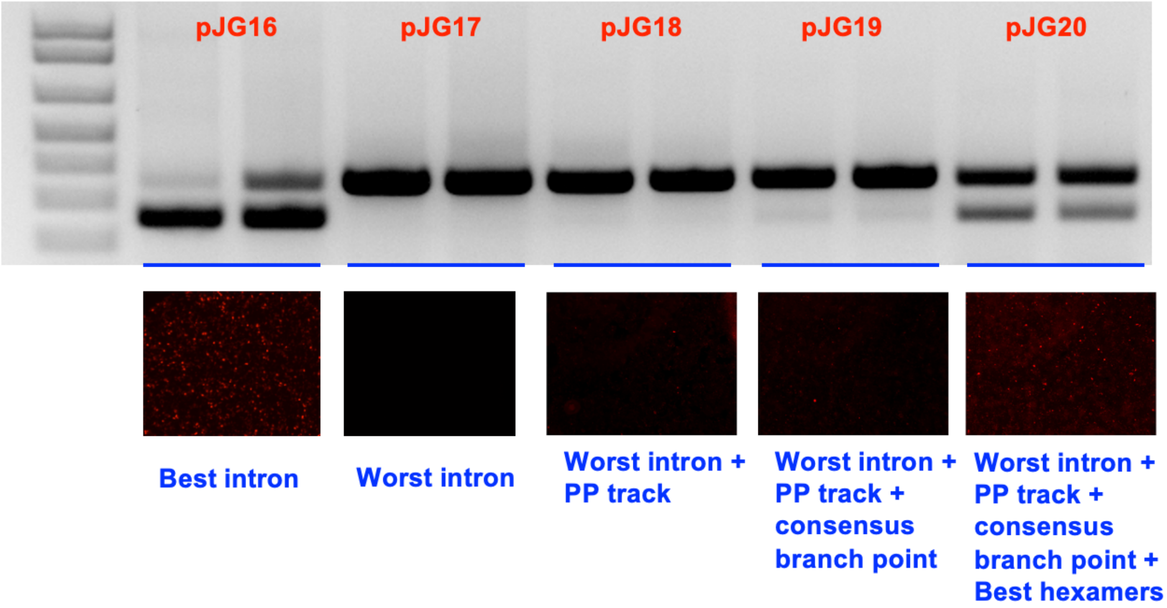

Supplementary Figure 23. Hexamers make a significant difference to splicing. The RT-PCR and fluorescence microscopy analysis of the best/worst synthetic introns along with modified constructs harbouring PP tract, branch point and the hexamers.
