# Supplementary material for: A basic framework to explain splice-site choice in eukaryotes
Source: Nat Commun. 2025 Sep 29;16:8284. doi: 10.1038/s41467-025-63622-9 (PMC12480690; doi:10.1038/s41467-025-63622-9)
Supplement: Supplementary file 2 — Description of Additional Supplementary Files [file 41467_2025_63622_MOESM2_ESM.pdf]

Description of Additional Supplementary Files

Supplementary Data

Supplementary Data 1. SpliSER-GWAS results for all associations detected in *Arabidopsis thaliana.* Only the highest and closest associated SNPs from the GWAS are shown.

Supplementary Data 2. SpliSER-GWAS results for all associations detected in *Drosophila melanogaster.* Only the highest and closest associated SNPs from the GWAS are shown.

Supplementary Data 3. SpliSER-GWAS results for all associations detected in the Humans. Only the highest and closest associated SNPs from the GWAS are shown.

Supplementary Data 4. Inferred best and worst nucleotides from the associated SNPs that are also closest to the splice-sites from GWAS data. Data for Arabidopsis, Drosophila and Humans and a combined analysis of all three species for donors and acceptors is given. Top rows represent the positions, and bottom row represents the inferred nucleotides.

Supplementary Data 5. The mean Splice-site Strength Estimate (SSE) of splice donor sites, with or without particular nucleotides in the -3 to +8 region. Downsampled in each species to 1-2 million site/sequence/strength combinations.

Supplementary Data 6. The mean Splice-site Strength Estimate (SSE) of splice acceptor sites, with or without particular nucleotides in the -7 to +3 region. Downsampled in each species to 1-2 million site/sequence/strength combinations.

Supplementary Data 7. The mean Splice-site Strength Estimate (SSE) of splice donor sites, with pairwise combinations of nucleotides at different positions in the -3 to +7 region, and their deviation

from an additive model. Downsampled in each species to 1-2 million site/sequence/strength combinations.

Supplementary Data 8. The mean Splice-site Strength Estimate (SSE) of splice acceptor sites, with pairwise combinations of nucleotides at different positions in the -7 to +3 region, and their deviation from an additive model. Downsampled in each species to 1-2 million site/sequence/strength combinations.

Supplementary Data 9. Constructs generated to test introns with various sequence combinations in this study.

Supplementary Data 10. Proportions of used and unused donor hexamers in human data.

Supplementary Data 11. Proportions of used and unused acceptor hexamers in human data

Supplementary Data 12. Donor hexamer counts, strengths, and ranks for all species. *Sc* - *Saccharomyces cerevisiae* baker's yeast, *Sp* - *Saccharomyces pombe* budding yeast, Algae – *Chlamydomonas*, *Chara* - Charaphycean algae, *Mp* - *Marchantia polymorpha* liverwort, *Os* -*Oryzae sativa* rice, *Zm* – *Zea mays* corn, Sorghum – Sorghum bicolor, *AT- Arabidopsis thaliana*, Canola – *Brassica napus*, Opium – Opium, Potato – potato, *Sl* – *Solanum lycopersicum* tomato, Sponge – sponges, *Hv* – *Hydra vulgaris*, *Ce* – *Caenorhabditis elegans* worm, *Dm* – *Drosophila melanogaster* flies, Octopus – octopus, *Dr* – *Danio rario* Zebrafish, *Xenopus* – *Xenopus* frog, Cobra – cobra, Chicken – chicken, *Mm* – *Mus musculus* Mouse, Pig – pig, *Pt* – *Pan troglodytes* Chimpanzee, *Hs* – *Homo sapiens* humans. For Arabidopsis, Drosophila and Humans, the rankings were calculated from all individuals that were part of the GWAS data. For all other species, a single transcriptome data was used to generate the rankings.

Supplementary Data 13. Acceptor hexamer counts, strengths, and ranks for all species. *Sc - Saccharomyces cerevisiae* baker's yeast, *Sp - Saccharomyces pombe* budding yeast, Algae – *Chlamydomonas*, *Chara* - Charaphycean algae, *Mp - Marchantia polymorpha* liverwort, *Os -Oryzae sativa* rice, *Zm – Zea mays* corn, Sorghum – Sorghum bicolor, *AT- Arabidopsis thaliana*, Canola – *Brassica napus*, Opium – Opium, Potato – potato, *Sl – Solanum lycopersicum* tomato, Sponge – sponges, *Hv – Hydra vulgaris*, *Ce – Caenorhabditis elegans* worm, *Dm – Drosophila melanogaster* flies, Octopus – octopus, *Dr – Danio rario* Zebrafish, *Xenopus – Xenopus* frog, Cobra – cobra, Chicken – chicken, *Mm – Mus musculus* Mouse, Pig – pig, *Pt – Pan troglodytes* Chimpanzee, *Hs – Homo sapiens* humans. For Arabidopsis, Drosophila and Humans, the rankings were calculated from all individuals that were part of the GWAS data. For all other species, a single transcriptome data was used to generate the rankings.

Supplementary Data 14. R^2 values of correlations between hexamer rankings of acceptors (A & B) and donors (C & D) based on counts (A & C) or splice-site strength (B & D) between diverse species.

Supplementary Data 15. PhenoScanner scan results for phenotypes associated with SNPs that are detected to be the highest associated SNPs in the SpliSER-GWAS analysis of the human heart atrial tissue RNA-seq data.
