# Supplementary material for: A basic framework to explain splice-site choice in eukaryotes
Source: Nat Commun. 2025 Sep 29;16:8284. doi: 10.1038/s41467-025-63622-9 (PMC12480690; doi:10.1038/s41467-025-63622-9)
Supplement: Supplementary file 4 — Reporting Summary [file 41467_2025_63622_MOESM4_ESM.pdf]

nature portfolio

Corresponding author(s): Sureshkumar Balasubramanian

Last updated by author(s): 2025-Jul-27

Reporting Summary

Nature Portfolio wishes to improve the reproducibility of the work that we publish. This form provides structure for consistency and transparency in reporting. For further information on Nature Portfolio policies, see our Editorial Policies and the Editorial Policy Checklist.

Statistics

For all statistical analyses, confirm that the following items are present in the figure legend, table legend, main text, or Methods section.

n/a	Confirmed	
☐	☒	The exact sample size (*n*) for each experimental group/condition, given as a discrete number and unit of measurement
☐	☒	A statement on whether measurements were taken from distinct samples or whether the same sample was measured repeatedly
☐	☒	The statistical test(s) used AND whether they are one- or two-sided<br>*Only common tests should be described solely by name; describe more complex techniques in the Methods section.*
☐	☒	A description of all covariates tested
☐	☒	A description of any assumptions or corrections, such as tests of normality and adjustment for multiple comparisons
☐	☒	A full description of the statistical parameters including central tendency (e.g. means) or other basic estimates (e.g. regression coefficient) AND variation (e.g. standard deviation) or associated estimates of uncertainty (e.g. confidence intervals)
☐	☒	For null hypothesis testing, the test statistic (e.g. *F*, *t*, *r*) with confidence intervals, effect sizes, degrees of freedom and *P* value noted<br>*Give P values as exact values whenever suitable.*
☒	☐	For Bayesian analysis, information on the choice of priors and Markov chain Monte Carlo settings
☒	☐	For hierarchical and complex designs, identification of the appropriate level for tests and full reporting of outcomes
☒	☐	Estimates of effect sizes (e.g. Cohen's *d*, Pearson's *r*), indicating how they were calculated

Our web collection on statistics for biologists contains articles on many of the points above.

Software and code

Policy information about availability of computer code

Data collection	SRA tool kit, Microsoft Excel
Data analysis	Arabidopsis RNA-seq data was aligned with TopHat2. Drosophila data was aligned with STAR version 2.7. Samtools version 1.12 was used to index fils and Regtools was used to generate junction files. SpliSER v0.1.8 was used for splicing analysis. GWAS was carried out with GEMMA v0.98.3. Peak calling was carried out using Manhattan Harverster. RNA binding motifs were analysed using ATtRACT database. Distance correlations were computed using MEGA. fastq-multx was used to analyse multiplexed data from Rosenberg et al.

For manuscripts utilizing custom algorithms or software that are central to the research but not yet described in published literature, software must be made available to editors and reviewers. We strongly encourage code deposition in a community repository (e.g. GitHub). See the Nature Portfolio guidelines for submitting code & software for further information.

Data

Policy information about availability of data

All manuscripts must include a data availability statement. This statement should provide the following information, where applicable:

- Accession codes, unique identifiers, or web links for publicly available datasets
- A description of any restrictions on data availability
- For clinical datasets or third party data, please ensure that the statement adheres to our policy

Arabidopsis RNA-seq Data is from the 1001 genome project and available under PRJNA319904. Drosophila RNA-seq data is from the Drosophila Genotype Reference

Panel 2 (DGRP2) project and the Human RNA-seq data is from the GTEx project. Source data for most of the figures are either present in Supplemental Data or provided as a source data file.

Research involving human participants, their data, or biological material

Policy information about studies with human participants or human data. See also policy information about sex, gender (identity/presentation), and sexual orientation and race, ethnicity and racism.

Reporting on sex and gender	This study did not involve direct recruitment of human participants or data generation from specific individuals. We used GTEx data that is deidentified and in general data was not segregated for sex and gender.
Reporting on race, ethnicity, or other socially relevant groupings	This study did not explore any data base on any groupings.
Population characteristics	N/A
Recruitment	This study did not involve any recruitment of human participants.
Ethics oversight	This study utilised restricted access data from the GTEx project and as such does not require ethics committee approval.

Note that full information on the approval of the study protocol must also be provided in the manuscript.

Field-specific reporting

Please select the one below that is the best fit for your research. If you are not sure, read the appropriate sections before making your selection.

☒ Life sciences ☐ Behavioural & social sciences ☐ Ecological, evolutionary & environmental sciences

For a reference copy of the document with all sections, see nature.com/documents/nr-reporting-summary-flat.pdf

Life sciences study design

All studies must disclose on these points even when the disclosure is negative.

Sample size	All samples sizes are given with appropriate figures/discussions.
Data exclusions	No data was excluded and any data filtering criteria is specified in the manuscript.
Replication	All data is publicly available and could be replicated with the descriptions in the methods.
Randomization	Study included controls and all controls are clearly defined in methods
Blinding	The deidentified data was used and thus the study in itself involved blinding

Reporting for specific materials, systems and methods

We require information from authors about some types of materials, experimental systems and methods used in many studies. Here, indicate whether each material, system or method listed is relevant to your study. If you are not sure if a list item applies to your research, read the appropriate section before selecting a response.

Materials & experimental systems

n/a	Involved in the study	
☒	☐	Antibodies
☐	☒	Eukaryotic cell lines
☒	☐	Palaeontology and archaeology
☐	☒	Animals and other organisms
☒	☐	Clinical data
☒	☐	Dual use research of concern
☐	☒	Plants

Methods

n/a	Involved in the study	
☒	☐	ChIP-seq
☒	☐	Flow cytometry
☒	☐	MRI-based neuroimaging

Eukaryotic cell lines

Policy information about cell lines and Sex and Gender in Research

Cell line source(s)	HEK293T commerically available cells were used (ATCC- CRL-3216).
Authentication	The cells were authenticated using sequencing of cDNAs that are tested for splicing differences.
Mycoplasma contamination	Tested by PCR
Commonly misidentified lines (See ICLAC register)	*Name any commonly misidentified cell lines used in the study and provide a rationale for their use.*

Animals and other research organisms

Policy information about studies involving animals; ARRIVE guidelines recommended for reporting animal research, and Sex and Gender in Research

Laboratory animals	Drosophila melanogaster
Wild animals	The RNA-seq data is from DGRP project, which involves
Reporting on sex	The DGRP data is for both male and female lines and the data has been reported for both sexes in Supplemental Data 2.
Field-collected samples	This study did not involve any field-collected samples.
Ethics oversight	No experiments involving live animals are reported in this manuscript and thus does not require ethical approval.

Note that full information on the approval of the study protocol must also be provided in the manuscript.

Dual use research of concern

Policy information about dual use research of concern

Hazards

Could the accidental, deliberate or reckless misuse of agents or technologies generated in the work, or the application of information presented in the manuscript, pose a threat to:

No	Yes	
☒	☐	Public health
☒	☐	National security
☒	☐	Crops and/or livestock
☒	☐	Ecosystems
☒	☐	Any other significant area

Experiments of concern

Does the work involve any of these experiments of concern:

No	Yes	
☒	☐	Demonstrate how to render a vaccine ineffective
☒	☐	Confer resistance to therapeutically useful antibiotics or antiviral agents
☒	☐	Enhance the virulence of a pathogen or render a nonpathogen virulent
☒	☐	Increase transmissibility of a pathogen
☒	☐	Alter the host range of a pathogen
☒	☐	Enable evasion of diagnostic/detection modalities
☒	☐	Enable the weaponization of a biological agent or toxin
☒	☐	Any other potentially harmful combination of experiments and agents

Plants

Seed stocks	Arabidopsis thaliana, Strains of 1001 genome project
Novel plant genotypes	There are no novel plant genotypes used in this study.
Authentication	The analysis of spliced products were verified by sequencing.
